# Endocytosis in the axon initial segment maintains neuronal polarity

**DOI:** 10.1038/s41586-022-05074-5

**Published:** 2022-08-17

**Authors:** Kelsie Eichel, Takeshi Uenaka, Vivek Belapurkar, Rui Lu, Shouqiang Cheng, Joseph S. Pak, Caitlin A. Taylor, Thomas C. Südhof, Robert Malenka, Marius Wernig, Engin Özkan, David Perrais, Kang Shen

**Affiliations:** 1 Howard Hughes Medical Institute, Department of Biology, Stanford University, Stanford, CA USA; 2 Department of Pathology, Stanford University School of Medicine, Stanford, CA USA; 3 Institute for Stem Cell Biology and Regenerative Medicine, Stanford University School of Medicine, Stanford, CA USA; 4 University of Bordeaux, CNRS, Interdisciplinary Institute for Neuroscience, Bordeaux, France; 5 Department of Molecular and Cellular Physiology, Stanford University, Stanford, CA USA; 6 Howard Hughes Medical Institute, Stanford University School of Medicine, Stanford, CA USA; 7 Nancy Pritzker Laboratory, Department of Psychiatry and Behavioral Sciences, Stanford University, Stanford, CA USA; 8 Department of Biochemistry and Molecular Biology, University of Chicago, Chicago, IL USA; 9 Grossman Institute of Neuroscience, Quantitative Biology and Human Behavior, University of Chicago, Chicago, IL USA

**Keywords:** Cellular neuroscience, Cell polarity, Membrane trafficking, Molecular neuroscience

## Abstract

Neurons are highly polarized cells that face the fundamental challenge of compartmentalizing a vast and diverse repertoire of proteins in order to function properly^1^. The axon initial segment (AIS) is a specialized domain that separates a neuron’s morphologically, biochemically and functionally distinct axon and dendrite compartments^2,3^. How the AIS maintains polarity between these compartments is not fully understood. Here we find that in *Caenorhabditis elegans*, mouse, rat and human neurons, dendritically and axonally polarized transmembrane proteins are recognized by endocytic machinery in the AIS, robustly endocytosed and targeted to late endosomes for degradation. Forcing receptor interaction with the AIS master organizer, ankyrinG, antagonizes receptor endocytosis in the AIS, causes receptor accumulation in the AIS, and leads to polarity deficits with subsequent morphological and behavioural defects. Therefore, endocytic removal of polarized receptors that diffuse into the AIS serves as a membrane-clearance mechanism that is likely to work in conjunction with the known AIS diffusion-barrier mechanism to maintain neuronal polarity on the plasma membrane. Our results reveal a conserved endocytic clearance mechanism in the AIS to maintain neuronal polarity by reinforcing axonal and dendritic compartment membrane boundaries.

## Abstract (web summary)

Endocytosis and degradation of plasma membrane proteins in the axon initial segment, together with the diffusion-barrier mechanism, maintain a polarized distribution of plasma membrane proteins in *Caenorhabditis elegans*, mouse, rat and human neurons.

## Main

Cellular compartmentalization is a fundamental feature of eukaryotic cells, which enables them to organize biochemical reactions and create functional specializations^4^. Neurons in particular are highly compartmentalized into distinct axonal and dendritic domains of the plasma membrane. To achieve this compartmentalization, neurons must sort and transport thousands of proteins to each domain and maintain their polarized distribution over the lifetime of the neuron^1^. This extreme polarization underlies neuronal function, and loss of neuronal polarity is associated with neurological dysfunction and neurodegenerative diseases^5–7^.

A critical region for neuronal polarity is the AIS, a specialized boundary zone that separates the axonal and somato-dendritic domains^2,8^. The AIS is molecularly defined by ankyrinG, the AIS master organizer, and by a dense submembranous cytoskeletal network^9,10^. The currently known functions of the AIS in neuronal polarity fall into two general categories: (1) intracellular sorting that regulates vesicle transport^3,11–18^, and (2) a diffusion barrier to slow the movement of axonal and dendritic proteins on the plasma membrane^19–21^. However, the mechanisms by which the AIS maintains the stringent compartmentalization of transmembrane proteins between the contiguous axonal and dendritic plasma membrane of the neuron remain unresolved. Here, we describe a distinct and active mechanism by which polarized transmembrane proteins are removed from the AIS plasma membrane through endocytosis and degraded to maintain their axonal or dendritic compartmentalization. We find that endocytic clearance of polarized receptors from the AIS membrane is conserved and used by diverse polarized receptors.

## Evidence of an AIS in *C. elegans*

**Fig. 1:**
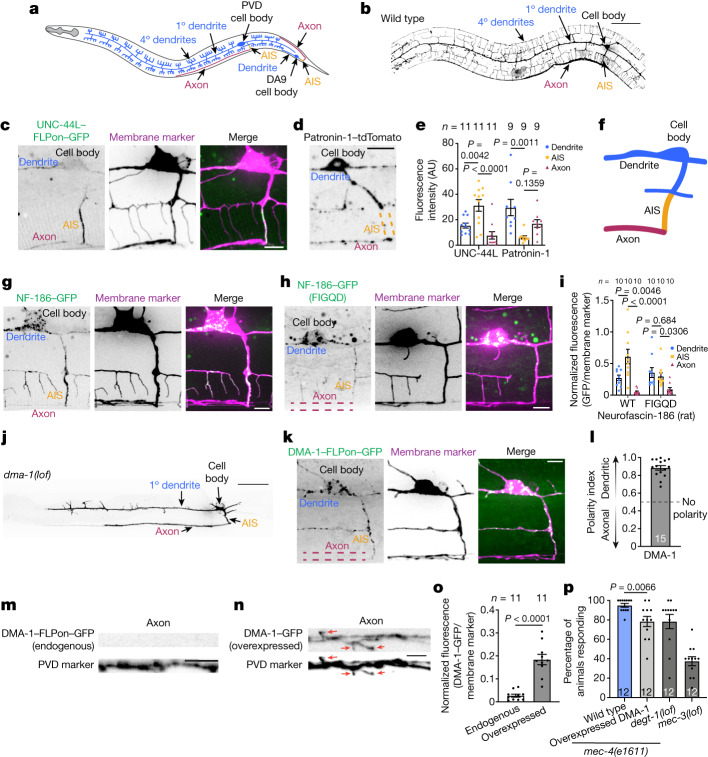
An in vivo intact animal system to study AIS function in neuronal polarity. **a**, Schematic of *C. elegans* PVD and DA9 neurons. 1°, primary; 4°, quaternary. **b**, Localization of the myristoylated GFP membrane marker in the wild-type PVD neuron. Scale bar, 50 µm. **c**, Cell-specific endogenous labelling of the long isoform of UNC-44 (UNC-44L–FLPon–GFP) using flippase-mediated recombination. Scale bar, 5 µm. **d**, Patronin-1–tdTomato localization in the PVD neuron. Scale bar, 10 µm. **e**, Quantification of UNC-44L and patronin-1 in PVD neuronal domains. AU, arbitrary units. **f**, Schematic of PVD neuronal domains. **g**, Rat NF-186 localization in the PVD neuron. Scale bar, 5 µm. **h**, Rat NF-186(FIGQD) localization in the PVD neuron. Scale bar, 5 µm. **i**, Average fluorescence of rat wild-type and mutant NF-186 proteins in PVD neuronal domains. **j**, Localization of a myristoylated GFP membrane marker of the PVD neuron in *dma-1(lof)* *C. elegans* animals. Scale bar, 50 µm. **k**,**l**, Cell-specific endogenous labelling (**k**) and polarity index (**l**) of DMA-1–FLPon–GFP in *C. elegans* animals. Scale bar, 10 µm. **m**,**n**, The axonal region of *C. elegans* animals expressing endogenous (**m**) or overexpressed (**n**) DMA-1–GFP. The same imaging conditions and formatting were used in both panels. Scale bars, 5 µm. Red arrows indicate aberrant axonal branches. **o**, Mean normalized GFP fluorescence in the axon of *C. elegans* animals described in **m**,**n**. **p**, Escape behaviour in *C. elegans* animals in response to a harsh touch stimulus. *C. elegans* animals carrying mutations in *degt-1* and *mec-3*, which encode a DEG/ENaC channel and a homeobox transcription factor that controls the differentiation of touch receptor neurons, respectively, were included as control animals with known behavioural defects. Data are shown as mean ± s.e.m. *n* represents the number of individual animals. **e**,**i** One-way ANOVA with Dunnett’s test. **o**, Two-tailed unpaired *t*-test with Welch’s correction. **p**, Two-tailed unpaired *t*-test. Source data

**Extended Data Fig. 1:**
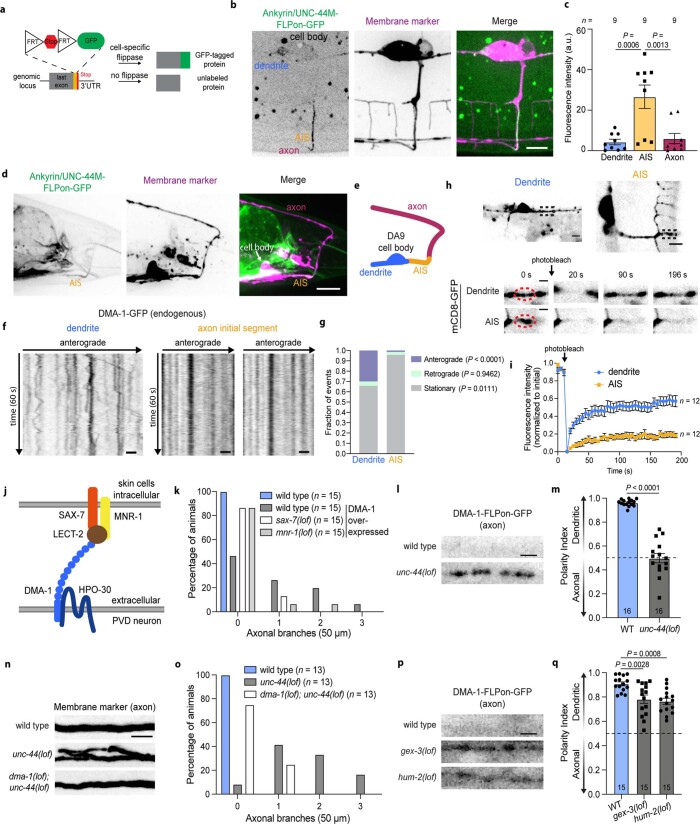
*C. elegans* neurons have hallmarks of an AIS. (**a**) Schematic of flippase-mediated GFP-tagging of proteins for cell-specific endogenous labeling. (**b**) Cell-specific endogenous expression of the medium isoform of ankyrin/UNC-44 (UNC-44M-FLPon-GFP) in the PVD neuron. (**c**) Average fluorescence intensity in each neuronal domain. (**d**) Cell-specific endogenous expression of UNC-44M-FLPon-GFP in the DA9 neuron. (**e**) Schematic of DA9 neuronal domains. (**f**) Kymograph analysis of endogenous DMA-1-GFP vesicular trafficking. (**g**) Quantification of DMA-1-GFP vesicular trafficking (n = 70 vesicles/region from 13 (dendrite) or 26 (AIS) animals over 6 experiments). (**h**) Confocal microscopy images of mCD8-GFP after fluorescence recovery after photobleaching (FRAP). Black squares indicate zoomed regions. (**i**) Fluorescence intensity of the red region in h. FRAP recovery kinetics are sensitive to neurite geometry, which may affect recovery rates. *P* *<* 0.0001 using a two-way ANOVA. (**j**) Schematic of the receptor complex mediating PVD dendrite branching. (**k**) Axonal branches in the 50 microns distal to the AIS. (**l**) Endogenous DMA-1-FLPon-GFP localization in the axon of the indicated genotypes. (**m**) DMA-1-FLPon-GFP polarity index of animals described in l. (**n**) Axonal region of animals of the indicated genotype. (**o**) Axonal branches in the 50 microns distal to the AIS. (**p**) Endogenous DMA-1-FLPon-GFP localization in the axon of the indicated genotypes. (**q**) DMA-1-FLPon-GFP polarity index of animals described in p. Data are shown as mean ± s.e.m. *N*-values are indicated on bar graphs and represent the number of animals. P values in c and q were calculated using a one-way ANOVA with Dunnett’s. P values in g were calculated with a mixed model ANOVA with Šidák multiple comparison test. P values in m were calculated using an unpaired two tailed t test with Welch’s correction. Scale bars, 10 µm (d), 5 µm (b, h top, n), and 2 µm (f, h bottom, l, p). Source data

To investigate AIS function in neuronal polarity, we developed the *C. elegans* PVD sensory neuron as an in vivo model to study neuronal polarity with single-cell resolution in living animals. The PVD neuron is exceptionally polarized and extends a highly branched dendrite and, by stark contrast, a single unbranched axon (Fig. 1a,b). We used this neuron to identify hallmarks of an AIS in *C. elegans* neurons. The *C. elegans* genome encodes a conserved ankyrin gene^22,23^ (*unc-44*). However, whether there is an AIS in *C. elegans* neurons has remained unclear owing to difficulties in determining the subcellular localization of the UNC-44 protein. To overcome these technical challenges, we used a flippase (FLP)-mediated cell-specific labelling strategy (Extended Data Fig. 1a) to endogenously label UNC-44 with GFP in the PVD neuron. We found that both the medium and the long isoforms of UNC-44 are enriched at the proximal end of the PVD axon (Fig. 1c and Extended Data Fig. 1b,c). Additionally, a microtubule minus-end-binding protein, patronin-1 (an orthologue of CAMSAP), was largely absent from the UNC-44-enriched region of the PVD neuron (Fig. 1d,e), consistent with observations of the AIS in vertebrate neurons^24^. Therefore, the PVD neuron has molecular hallmarks of a vertebrate AIS after the last dendritic branch and before the axon enters the ventral nerve cord (Fig. 1f). We also found evidence of an AIS in other *C. elegans* neurons, such as the bipolar DA9 motor neuron that has an enrichment of UNC-44 at the proximal end of its axon (Fig. 1a and Extended Data Fig. 1d,e).

To test the molecular conservation of the *C. elegans* AIS architecture, we expressed a rat isoform of neurofascin-186 (NF-186), an AIS-resident protein that is enriched at the AIS through a FIGQY ankyrinG-binding motif^9,25,26^. We found that wild-type rat NF-186 is enriched at the AIS of the PVD neuron, whereas a mutant version of NF-186 that does not bind ankyrinG^25^ (NF-186(FIGQD)) is not enriched at the AIS (Fig. 1g–i). We also found functional evidence of the AIS as both an intracellular vesicle filter and a diffusion barrier by examining vesicular trafficking and membrane protein dynamics, respectively, in the dendrite versus the AIS (Extended Data Fig. 1f–i and Supplementary Videos 1 and 2). On the basis of these results, we consider the axonal segment delimited by the last dendritic branch and the point of entry into the ventral nerve cord to be a bona fide AIS with conserved molecular components and similar functions as in vertebrate systems (Fig. 1f).

Having established that *C. elegans* neurons have an AIS, we next investigated its role in establishing and maintaining neuronal polarity. We initially focused on the compartmentalization of the DMA-1 transmembrane receptor because it has a key role in establishing morphological polarity by mediating the extensive dendritic branching of the PVD neuron^27^ (Fig. 1j and Extended Data Fig. 1j). To study DMA-1 compartmentalization, we used single-cell endogenous labelling of DMA-1 in the intact *C. elegans* animal. Consistent with its branching function, endogenous DMA-1 was highly polarized to the dendrite and excluded from the axon (Fig. 1k–m). However, overexpression of DMA-1 increased its fluorescence in the dendrite as expected, but also caused DMA-1 to enter the axon (Fig. 1n). DMA-1 mislocalization to the axon caused aberrant axonal branching that was dependent on the same ligands that activate DMA-1 in the dendrite, demonstrating that a loss of DMA-1 molecular polarity causes morphological polarity defects in the PVD neuron (Extended Data Fig. 1j,k). Thus, the mechanisms that control DMA-1 compartmentalization are easily overwhelmed by its overexpression.

We next investigated the roles of known proteins in AIS function. A loss-of-function mutation in UNC-44, a large AIS scaffold molecule that is required for neuronal polarity^28^, caused a complete loss of DMA-1 dendritic polarity and induced the formation of aberrant axonal branches that were dependent on DMA-1 (Extended Data Fig. 1l–o). Filamentous actin and non-muscle myosin Va at the AIS are important for maintaining neuronal polarity^14,29,30^. Consistently, loss-of-function mutations in *gex-3*, a WAVE complex component that mediates branched filamentous actin^31^, and *hum-2*, an orthologue of myosin Va that mediates vesicular trafficking in the AIS^14,29,30^, both caused a partial loss of DMA-1 dendritic polarity (Extended Data Fig. 1p,q). We then examined behavioural consequences of a loss of polarity by using a harsh touch escape behavioural assay that provides a readout of PVD neuronal function^32^. We found that overexpression of DMA-1, which caused mislocalization of DMA-1 to the axon and aberrant axonal branches (Fig. 1n,o), was also associated with a behavioural deficit in response to a harsh touch stimulus, indicative of a loss of cellular function (Fig. 1p). Therefore, the strict dendritic polarization of DMA-1 is critical for proper neuronal function.

## AIS endocytosis of dendritic receptors

**Fig. 2:**
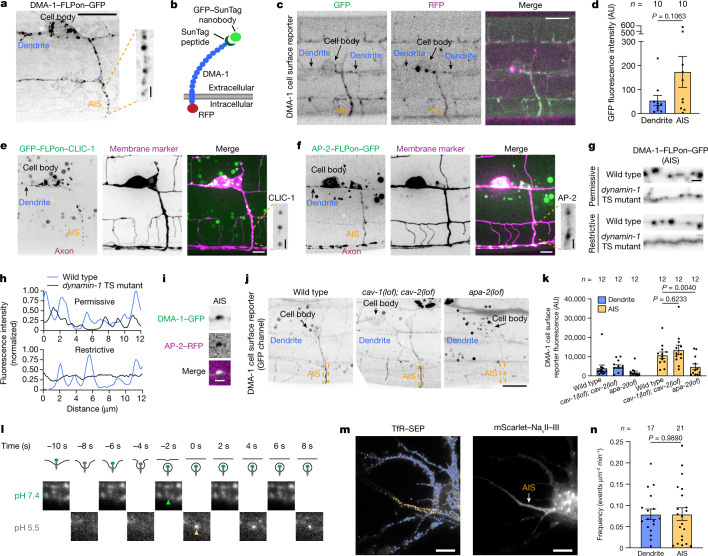
Endocytosis of dendritically polarized receptors in the AIS. **a**, Endogenous DMA-1–FLPon–GFP in the *C. elegans* PVD neuron AIS. Scale bar, 10 µm (main image), 1 µm (expanded selection). **b**, Schematic of the DMA-1 cell-surface reporter assay. DMA-1 is labelled with RFP and a 4× SunTag peptide. GFP-tagged SunTag nanobody is secreted from adjacent muscle cells. **c**,**d**, Confocal images (**c**) and DMA-1 cell-surface reporter fluorescence (**d**). Scale bar, 10 µm. **e**,**f**, Cell-specific endogenous expression of clathrin light chain (GFP–FLPon–CLIC-1) (**e**) and AP-2–FLPon–GFP (**f**). Asterisk indicates unrelated gut autofluorescence. Scale bar, 10 µm (main images), 1 µm (expanded selection). **g**, DMA-1–FLPon–GFP in the AIS of wild-type or *dynamin-1* temperature-sensitive (TS) *C. elegans* animals. Scale bar, 1 µm. **h**, Line scan analysis of DMA-1 from images in **g**. **i**, Endogenous DMA-1–GFP is concentrated into AP-2-labelled structures in the AIS. Scale bar, 1 µm. **j**, GFP signal from the DMA-1 cell-surface reporter. Scale bar, 10 µm. **k**, GFP fluorescence of the DMA-1 cell-surface reporter in *C. elegans* animals. **l**, Top, illustration of endocytosis and the SEP signal during the pulsed-pH protocol (grey represents quenching). Endocytic vesicle scission generates an acid-resistant fluorescent punctum in the subsequent pH 5.5 step. Bottom, AIS of a cultured rat neuron (DIV9) imaged during the pulsed-pH protocol. An endocytic event (yellow arrow) is indicated by a pH 5.5-resistant signal that corresponds to a pre-existing cluster at pH 7.4 (green arrow). Contrast is increased for the pH 5.5 frames. **m**, A transfected rat neuron in culture (DIV9). Crosses represent endocytic events detected during a 10-min pulsed-pH protocol in the AIS (yellow, 85 events) and other neuronal regions (blue, 797 events). Scale bars, 10 µm. **n**, Frequencies of events in the indicated region. Data are mean ± s.e.m. *n* represents the number of animals or cells. **k**, Two-way ANOVA with Šidák's multiple comparison test. **d**,**n**, Two-tailed unpaired *t*-test. Source data

**Extended Data Fig. 2:**
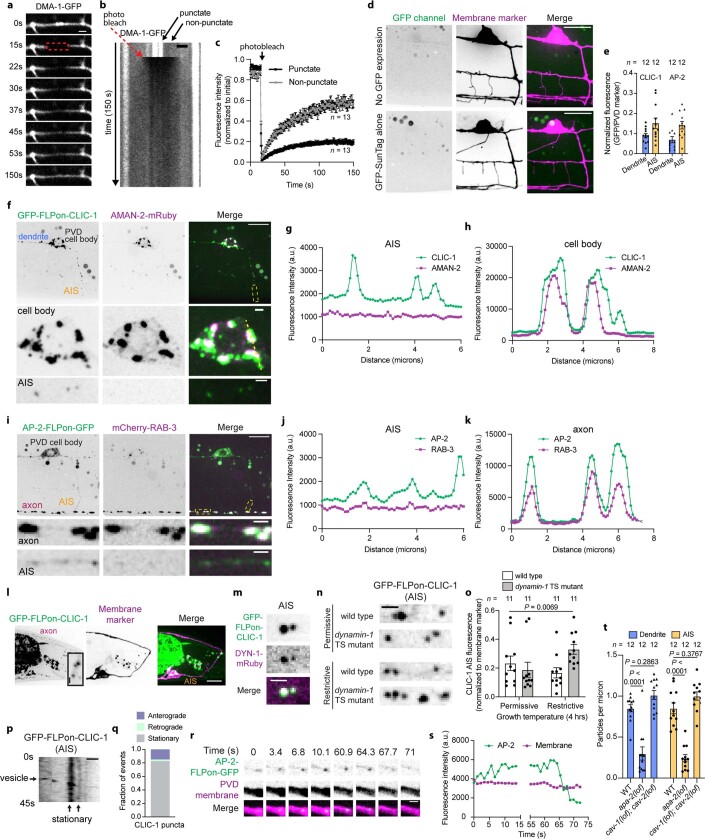
Characterization of endocytic vesicles in the AIS. (**a**) FRAP of DMA-1-GFP pools in the PVD dendrite. (**b**) Kymograph analysis of FRAP experiment in a. (**c**) FRAP analysis of DMA-1-GFP pools (*n* = 13 animals/condition; *P* *<* 0.0001 using a two-way ANOVA). (**d**) Confocal images of DMA-1 cell surface assay controls in PVD. (**e**) Quantification of cell-specific endogenous proteins. (**f**) Cell specific expression of endogenous GFP-FLPon-CLIC-1 and Golgi-localized alpha mannosidase/AMAN-2[1-84aa]-mRuby in PVD. The Golgi network contains several Golgi-stacks^66^. Linescan analysis of fluorescence intensity in the (**g**) AIS and **(h**) cell body from the regions marked by yellow in **f**. (**i**) AP-2-FLPon-GFP and mCherry-RAB-3 expression in PVD. Linescan analysis of fluorescence intensity in the (**j**) AIS and (**k**) axon from the regions marked by yellow in **i**. (**l**) Cell-specific endogenous expression of GFP-FLPon-CLIC-1 in DA9. Inset shows AIS zoom. (**m**) Cell-specific endogenous GFP-FLPon-CLIC-1 and dynamin-1-mRuby colocalize in the AIS. (**n**) Cell-specific endogenous GFP-FLPon-CLIC-1 puncta in the AIS of the indicated animals. (**o**) Average fluorescence intensity of experiments described in **n**. (**p**) Kymograph of cell-specific endogenous GFP-FLPon-CLIC-1 in the PVD AIS. (**q**) GFP-FLPon-CLIC-1 puncta dynamics in the AIS (*n* = 40 vesicles). (**r**) Montage and (**s**) fluorescence intensity of AP-2-FLPon-GFP in the AIS. (**t**) GFP-positive puncta density from the DMA-1 cell surface reporter assay in the AIS of the indicated animals. Data are shown as mean ± s.e.m. *N*-values are indicated on bar graphs and represent the number of animals. *P* values were calculated as follows: **o** using a Brown-Forsythe and Welch one-way ANOVA, and **t** using two-way ANOVA with Tukey’s multiple comparisons test. Scale bars, 10 µm (**d**, **f** top, **i** top, **l**), 2 µm (**a**, **b**, **m**, **n**), 1 µm (**f** middle, **f** bottom, **i** middle, **i** bottom,** l** inset, **p**, **r**). Source data

Endogenous DMA-1 was highly polarized to the dendrite and localized to multiple puncta in the dendrite and the AIS (Figs. 1k, l and 2a). This observation motivated us to investigate compartmentalization mechanisms for DMA-1. We first investigated the properties of DMA-1 puncta using fluorescence recovery after photobleaching (FRAP). We reasoned that DMA-1 in intracellular vesicles would have different recovery profiles compared with DMA-1 on the plasma membrane, which can recover through lateral diffusion on the contiguous plasma membrane. Indeed, we found evidence for two pools of DMA-1 with distinct dynamics: a non-punctate pool and a punctate pool with a larger immobile fraction compared to the non-punctate pool (Extended Data Fig. 2a–c). Thus, the punctate pool of DMA-1 is likely to represent intracellular vesicles, which are unable to recover owing to being predominantly in isolated vesicles.

We then sought to determine the origin of dendritic protein-containing vesicles that were present in the AIS. We considered two possibilities: DMA-1-containing vesicles could represent either biosynthetic or endolysosomal vesicles. To distinguish between these possibilities, we used a DMA-1 cell-surface reporter assay in which DMA-1 is tagged with an extracellular SunTag peptide and an intracellular RFP, and a SunTag nanobody fused to GFP is secreted from the surrounding muscle cells (Fig. 2b). This assay distinguishes DMA-1 puncta in biosynthetic compartments (red only) from DMA-1 that has been exposed to the cell surface (red and green). Red-only puncta were visible in the cell body, whereas the AIS contained a strong GFP signal that represented specific binding of the GFP SunTag nanobody to the GFP SunTag peptide, based on the requirement of the GFP SunTag peptide for a visible GFP signal (Fig. 2c,d and Extended Data Fig. 2d). Thus, the AIS contained a pool of DMA-1 that was either currently or previously on the plasma membrane before incorporation into the endolysosomal system and cannot be accounted for solely by the biosynthetic pathway.

Based on the dominant pool of puncta containing cell-surface-exposed DMA-1 in the AIS and the observation that DMA-1 puncta rarely pass through the AIS (Extended Data Fig. 1f,g), we considered whether dendritic protein-containing vesicles were generated in the AIS itself through endocytosis. We performed a series of experiments to investigate DMA-1 endocytosis within the AIS. We first examined the subcellular localization of endogenously labelled core components of clathrin-mediated endocytosis. We found that clathrin light chain (CLIC-1), a coat protein of clathrin-coated pits and adapter protein-2 (AP-2 (called APA-2 in *C. elegans*)), a clathrin-associated endocytic adapter protein^33^, both formed diffraction-limited puncta in the AIS (Fig. 2e,f and Extended Data Fig. 2e). CLIC-1 was also present on large structures in the cell body that overlapped with the Golgi network marker AMAN-2, and AP-2 colocalized with the synaptic vesicle marker RAB-3 in the axon, consistent with their known functions at the Golgi and presynaptic terminal, respectively^34,35^ (Extended Data Fig. 2f–k). We also found CLIC-1 puncta in the AIS of the *C. elegans* DA9 motor neuron (Extended Data Fig. 2l). Consistent with our light microscopy results, clathrin-coated pits have been reported in electron microscopy studies of the AIS^36^, and AP-2 has been shown to be enriched in the AIS using proximity-label mass spectrometry^18^.

Several lines of evidence demonstrate that the CLIC-1 puncta are functional endocytic clathrin-coated structures. First, they were co-labelled with dynamin-1, the GTPase that pinches endocytic vesicles from the plasma membrane (Extended Data Fig. 2m). Further, the intensity of CLIC-1 puncta was increased in *dynamin-1* temperature-sensitive mutants, indicating that their internalization and disappearance is dependent on dynamin-1 function (Extended Data Fig. 2n,o). More than 80% of CLIC-1 puncta in the AIS were stationary and thus were not mobile transport vesicles transiently passing through the AIS (Extended Data Fig. 2p,q and Supplementary Video 3). We also observed the gradual formation and sudden disappearance of AP-2-labelled puncta, consistent with the dynamics of clathrin-mediated endocytosis^37^ (Extended Data Fig. 2r,s and Supplementary Video 4).

We then sought to determine whether DMA-1 engages the clathrin-mediated endocytic machinery in the AIS. First, we found that DMA-1 puncta were generated rapidly in a dynamin-dependent manner in the AIS. Acute dynamin-1 inhibition resulted in a loss of DMA-1 puncta in the AIS (Fig. 2g,h). Then, we found that DMA-1 in the AIS was concentrated into AP-2-labelled structures, which have been used as a marker for clathrin-mediated endocytic events^38^ (Fig. 2i). Consistent with this, loss of *apa-2* function, but not *caveolin-1* (*cav-1*) and *caveolin-2* (*cav-2*) (major components of caveolar endocytosis), caused a decrease in the number of GFP-positive puncta in the AIS and a decrease in the overall GFP signal of the DMA-1 cell-surface reporter (Fig. 2j,k and Extended Data Fig. 2t). This suggests that the GFP signal from the DMA-1 cell-surface reporter largely reflects internalized receptors. Together, these results support that DMA-1 undergoes clathrin-mediated endocytosis in the AIS.

## Conservation of AIS endocytosis

**Extended Data Fig. 3:**
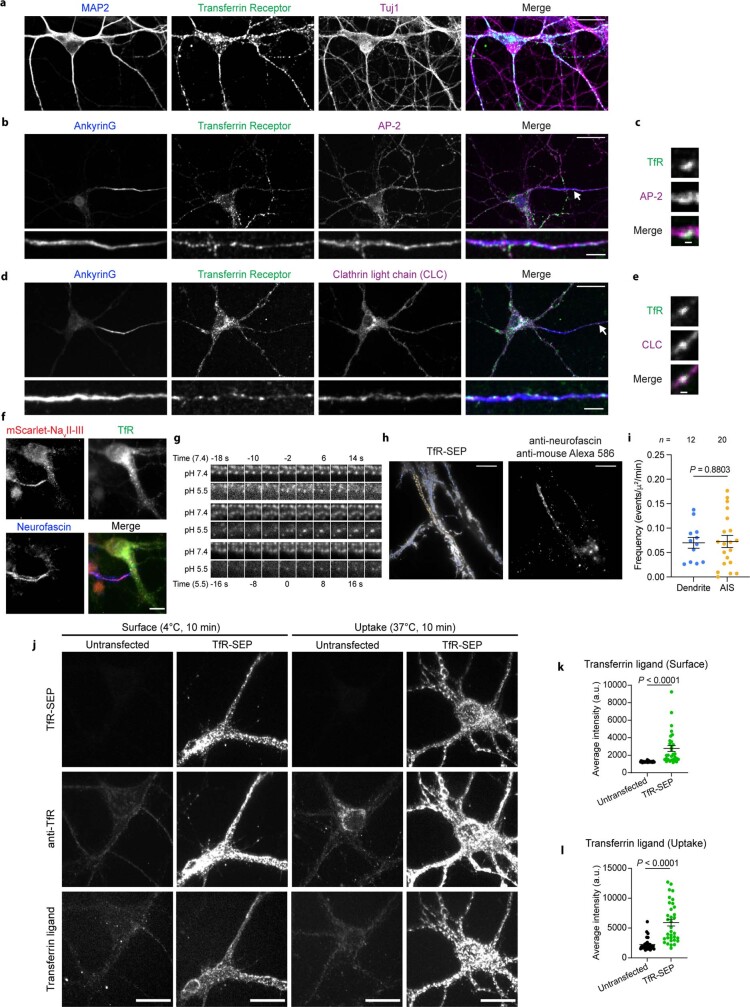
Endocytosis of dendritic receptors in the AIS of cultured mouse and rat neurons. (**a**, **b**) Confocal images of mouse neurons fixed and stained at DIV14 for the indicated endogenous proteins, including MAP2 (dendrites) and Tuj1 (all neurites). AIS zoom is shown on the bottom, and brightness is increased for better visibility. (**c**) AP-2 labelled structure containing TfR from the AIS of the image shown in **b** (white arrow). Brightness is increased for better visibility. (**d)** Confocal images of mouse neurons fixed and stained at DIV14 for the indicated endogenous proteins. AIS zoom is shown on the bottom, and brightness is increased for better visibility. (**e**) Clathrin-coated structure containing TfR in the AIS of the image shown in **d** (white arrow). Brightness is increased for better visibility. (**f**) Neurons transfected with TfR-SEP and mScarlet-Na_v_II-III, fixed at DIV10, and stained with anti-neurofascin (NF) antibody (clone A12/18) and anti-mScarlet antibody. Intense staining of both mScarlet-Na_v_II-III and NF defines the AIS. 18/20 transfected neurons (from 3 cultures) showed colocalization with endogenous NF. (**g**) Scission/endocytic example events from Fig. 2m, as defined as the appearance of a TfR-SEP cluster (i.e. an endocytic vesicle) at pH 5.5 (time 0) and corresponding to a preexisting cluster at pH 7.4 (time −2). Note that these vesicles remain visible at pH 5.5 for 3 frames or more. Contrast is increased for pH 5.5 frames for better visibility. (**h**) Same analysis as Fig. 2m for neurons transfected with TfR-SEP and incubated with anti-NF antibody (clone A12/18). (**i**) Frequencies of endocytic events in the AIS (20 recordings from 4 different cultures) and selected dendrites (12 recordings). Event frequency is similar between both AIS visualization methods: for neurons expressing mScarlet-Na_v_II-III, the frequency is 0.080 ± 0.015 events/μm^2^/min (*n *= 21) and for neurons stained with anti-NF antibody, the frequency is 0.073 ± 0.012 events/μm^2^/min (*n* = 20); *P* = 0.72 using an unpaired t test. (**j)** Hippocampal neurons (untransfected and TfR-SEP transfected) with surface bound or internalized transferrin ligand labelled with Alexa647, fixed and labelled with anti-transferrin receptor antibody (anti-TfR, clone H68.4). **(k**) Fluorescence intensity of surface transferrin ligand labeled with Alexa647 bound at 4 °C (surface) is 2.19 fold higher in TfR-SEP transfected neurons: 1266 ± 12 arbitrary units (a.u). in control (*n* = 32) versus 2773 ± 341 a.u. in TfR-SEP transfected neurons (*n* = 30) across 3 cultures. (**l)** Fluorescence intensity of internalized transferrin ligand labelled with Alexa647 is 2.64 fold higher in TfR-SEP transfected neurons: Control (2238 ± 174 a.u., *n* = 33) versus TfR-SEP (5911 ± 568 a.u., *n* = 34) across 3 cultures. *P* values in** i**, **k**, and **l** were calculated with a two-tailed unpaired *t* test. All data are shown as mean ± s.e.m. *N*-values are indicated on bar graphs and represent the number of neuronal regions scored for each condition. Scale bars, 20 µm (**a**, **b** top, **d** top, **j**), 10 µm (**f**, **h**), 5 µm (**b** bottom, **d** bottom) and 1 µm (**c**, **e**). Source data

To directly visualize endocytosis of dendritic receptors in the AIS and to test whether AIS endocytosis of dendritic receptors is conserved, we examined the transferrin receptor (TfR) in cultured mouse and rat neurons. The TfR is a prototypical dendritically polarized receptor that has been used to uncover many principles of AIS function in neuronal polarity^14,16,29,39^. We found that the dendritically polarized TfR is also present in the AIS of cultured mouse neurons and overlaps with AP-2 and clathrin light chain, components of clathrin-mediated endocytic machinery (Extended Data Fig. 3a–e). We then used a pulsed-pH protocol to resolve TfR-containing endocytic events in real time^40^. In this assay, the TfR is extracellularly tagged with the pH sensitive fluorophore super-ecliptic pHluorin (SEP), and endocytic events render SEP-labelled receptors resistant to the extracellular application of a membrane-impermeant acidic buffer. Therefore, by alternating the pH of the imaging buffer between pH 5.5 and 7.4 at 0.5 Hz during imaging, TfR–SEP-containing endocytic events were resolved as acid-resistant fluorescent puncta that abruptly appeared in the pH 5.5 imaging frame (Fig. 2l). We visualized the AIS in living cultured rat neurons using two labelling methods: (1) a genetically encoded construct expressing a voltage-gated sodium channel intracellular domain fused to mScarlet (mScarlet–Na_v_II–III) that is known to localize to the AIS and (2) incubation with a neurofascin antibody that detects the extracellular domain of neurofascin^41^ (Extended Data Fig. 3f). We observed TfR endocytic events in the somato-dendritic region, as expected^42^; however, we also observed robust TfR endocytosis in the AIS (Fig. 2l,m and Extended Data Fig. 3g,h). Notably, despite being dendritically polarized, the TfR undergoes endocytosis at the same frequency in the AIS as in the dendrite (Fig. 2n and Extended Data Fig. 3i), whereas we detected few TfR endocytic events in the axon distal to the AIS (Fig. 2m). Of note, AIS endocytosis is unlikely to be owing to exogenous expression of TfR–SEP because we found similar ratios of surface-bound and internalized transferrin ligand in TfR-SEP-expressing and untransfected cells (Extended Data Fig. 3j–l). Thus, transgenic TfR–SEP behaved similarly to endogenous TfR. Directly visualizing TfR endocytosis in the AIS distinguishes endocytic removal of TfR from the plasma membrane from known TfR intracellular sorting events at the AIS^14,16,29,39^.

**Extended Data Fig. 4:**
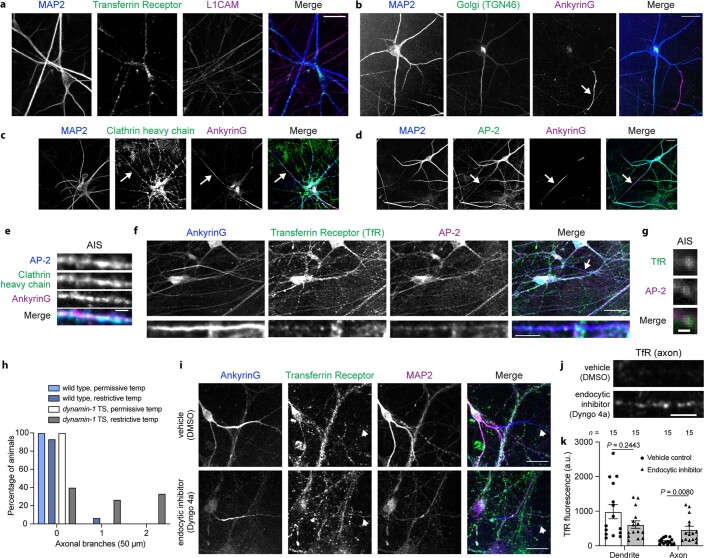
Endocytosis of dendritic receptors in the AIS of human neurons. (**a**) Dendritically polarized TfR and axonal L1CAM are polarized to their appropriate domains in induced human neurons that were fixed and stained at DIV26. (**b**) Induced human neurons have a single ankyrinG-labelled AIS when co-cultured with glial cells. (**c**, **d**) Confocal images of human neurons fixed and stained at DIV26 for endocytic proteins. Arrows designate the AIS. (**e**) AIS of induced human neurons that were fixed and stained at DIV26 for ankyrinG, AP-2, and clathrin heavy chain. (**f**) Confocal images of human neurons fixed and stained at DIV26 for the indicated endogenous proteins. Bottom images show the AIS (brightness is increased for better visibility). Note that extra-neuronal fluorescence represents endogenous staining in co-cultured glial cells. (**g**) Zoomed in region from **f** of an endocytic vesicle in the AIS. Brightness is increased for better visibility. (**h**) Histogram analysis of axonal branches in the 50 microns distal to the AIS in the indicated animals (*n* = 15 animals/genotype). (**i**) Confocal images of cultured mouse neurons pretreated with vehicle control (top, DMSO) or endocytic inhibitor (bottom, Dyngo 4a) for 5 h prior to being fixed and stained at DIV14. Arrows designate the axon. (**j**) Endogenous TfR fluorescence in the axon from neurons described in **i**. Arrows designate the cropped region. (**k**) Average TfR fluorescence in the axon compared to the dendrite. All data are shown as mean ± s.e.m. *N*-values are indicated on bar graphs and represent the number of individual cells scored for each condition. *P* values in **k** were calculated using a two-way ANOVA with Šidák multiple comparison test. Scale bars, 20 µm (**a**, **b**, **c**, **d**, **f** top, **i**), 5 µm (**f** bottom, **j**), 2 µm (**e**), 1 µm (**g**). Source data

We also found evidence of TfR endocytosis in the AIS of induced human neurons. These neuronal cultures were properly polarized (as indicated by the localization of the TfR to the dendrite and L1CAM to the axon^43^), had a single ankyrinG-labelled AIS that is known to exhibit AIS plasticity when co-cultured with glia^44^, and had clathrin-mediated endocytic machinery in the AIS (Extended Data Fig. 4a–e). Further, the TfR was present in the AIS and overlapped with AP-2-labelled endocytic structures (Extended Data Fig. 4f,g). Together, these data suggest that dendritic receptor endocytosis in the AIS is conserved from *C. elegans* to humans.

## Function of endocytosis in the AIS

**Fig. 3:**
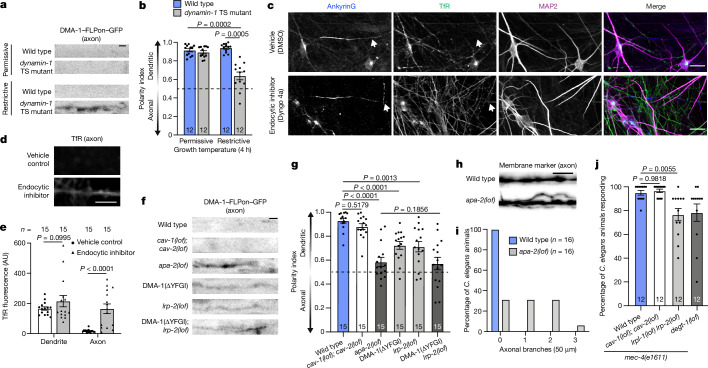
Endocytosis maintains dendritic receptor compartmentalization and is critical for neuronal function. **a**, Localization of endogenously labelled DMA-1–FLPon–GFP in the axon of wild-type or *dynamin-1* temperature-sensitive *C. elegans* animals. Scale bar, 2 µm. **b**, DMA-1–FLPon–GFP polarity index of *C. elegans* animals described in **a**. **c**, Confocal images of DIV26 human neurons treated with vehicle control (DMSO) or the endocytosis inhibitor Dyngo 4a for 18 h prior to fixation and staining for the indicated endogenous proteins. Arrows indicate axonal regions. Scale bars, 20 µm. **d**, TfR fluorescence in the axon of neurons described in **c**. Scale bars, 5 µm. **e**, Average TfR fluorescence intensity in the dendrite and axon of neurons described in **c**. **f**, Localization of endogenous DMA-1–FLPon–GFP in axons of wild-type and endocytic mutant *C. elegans* animals. Scale bar, 2 µm. **g**, DMA-1–FLPon–GFP polarity index of *C. elegans* animals described in **f**. **h**, A PVD axon labelled with a myristoylated GFP membrane marker in *C. elegans* animals of the indicated genotype. Scale bars, 5 µm. **i**, Number of axonal branches in the 50 µm distal to the AIS in *C. elegans* animals of the indicated genotype. **j**, Escape behaviour in *C. elegans* animals in response to a harsh touch stimulus. Wild-type and *degt-1* data are from Fig. 1p. Data are mean ± s.e.m. *n* represents the number of individual animals or cells for each condition. **b**,**e**, Two-way ANOVA with Šidák's multiple comparison test. **g**, Brown–Forsythe and Welch one-way ANOVA with Dunnett's test. **j**, One-way ANOVA with Dunnett’s test. Source data

Because endocytosis inherently removes transmembrane receptors from the plasma membrane, we considered the possibility that endocytosis in the AIS functions to remove polarized receptors from the membrane and thus functions in maintaining their axon–dendrite compartmentalization. Therefore, we first examined the consequences of global endocytic inhibition by perturbing dynamin function. Endocytic inhibition using a *dynamin-1* temperature-sensitive mutation caused a loss of DMA-1 dendritic polarity and defects in neuronal morphological polarity in the *C. elegans* PVD neuron (Fig. 3a,b and Extended Data Fig. 4h). In cultured mouse and human neurons, we found that endocytosis contributed to the dendritic compartmentalization of the TfR. Endocytic inhibition by treatment with Dyngo 4a caused an increase of TfR in the axon, thus weakening its polarized dendritic localization, although TfR was still enriched in the dendrite, indicating that a component of its dendritic targeting is independent of endocytosis (Fig. 3c–e and Extended Data Fig. 4i–k). These results demonstrate that endocytosis contributes to the polarized distribution of dendritic receptors.

**Fig. 4:**
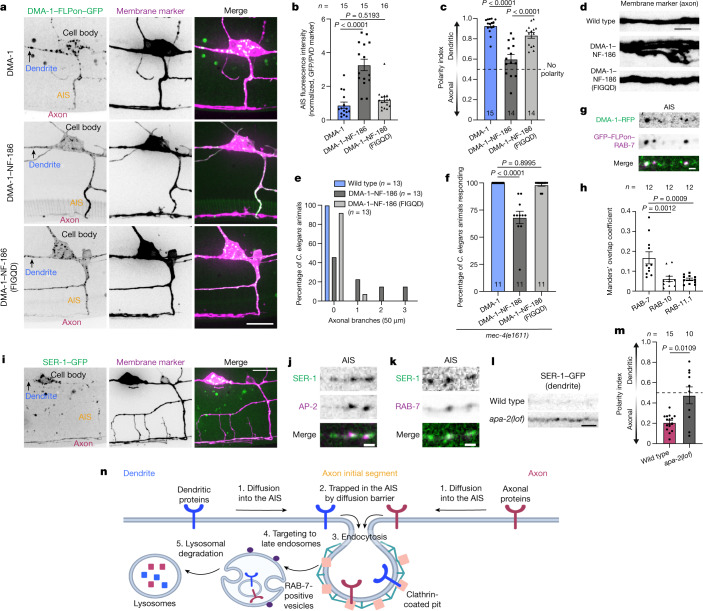
Antagonizing DMA-1 AIS endocytosis and identifying dendritic receptor post-endocytic targeting to RAB-7 positive late endosomes. **a**, Localization of endogenous DMA-1–FLPon–GFP and DMA-1–NF-186–FLPon–GFP chimeras in the *C. elegans* PVD neuron. Scale bar, 10 µm. **b**,**c**, AIS fluorescence (**b**) and polarity index (**c**) of chimaeras from *C. elegans* animals described in **a**. **d**, Axonal region of *C. elegans* animals expressing DMA-1–NF-186 chimaeras. Scale bar, 5 µm. **e**, Axonal branches in the 50 µm distal to the AIS in *C. elegans* animals described in **d**. **f**, Escape behaviour in *C. elegans* animals expressing DMA-1-NF-186 chimaeras in response to a harsh touch stimulus. **g**, Endogenous DMA-1–RFP is concentrated into GFP–FLPon–RAB-7 positive puncta in the AIS of the *C. elegans* PVD neuron. Scale bar, 1 µm. **h**, Manders’ overlap coefficient analysis for endogenous DMA-1 and endogenous Rab proteins in the PVD neuron. **i**, SER-1–GFP localization in the PVD neuron. Scale bar, 10 µm. **j**,**k**, SER-1–GFP is concentrated into AP-2-labelled clathrin-coated structures (**j**) and RAB-7-labelled late endosomes in the AIS (**k**) of the PVD neuron. Scale bars, 1 µm. **l**, SER-1–GFP localization in the dendrite of wild-type and *apa-2* mutant animals. Scale bar, 2 µm. **m**, SER-1–GFP polarity index for *C. elegans* animals described in **l**. **n**, A model depicting the steps of polarized receptor endocytic clearance from the AIS. Axonal (red) and dendritic (blue) transmembrane proteins: (1) diffuse laterally into the AIS; (2) are trapped in the AIS by a diffusion barrier; (3) are captured for endocytosis by binding the clathrin-mediated endocytic machinery; (4) are targeted to RAB-7-positive late endosomes; and (5) are degraded through lysosomal pathways. Model created with BioRender.com. Data are mean ±s.e.m. *n* represents the number of individual *C. elegans* animals for each condition. **b**,**c**,**f**,**h**, One-way ANOVA with Dunnett’s test. **m**, Two-tailed unpaired *t*-test with Welch’s correction. Source data

**Extended Data Fig. 6:**
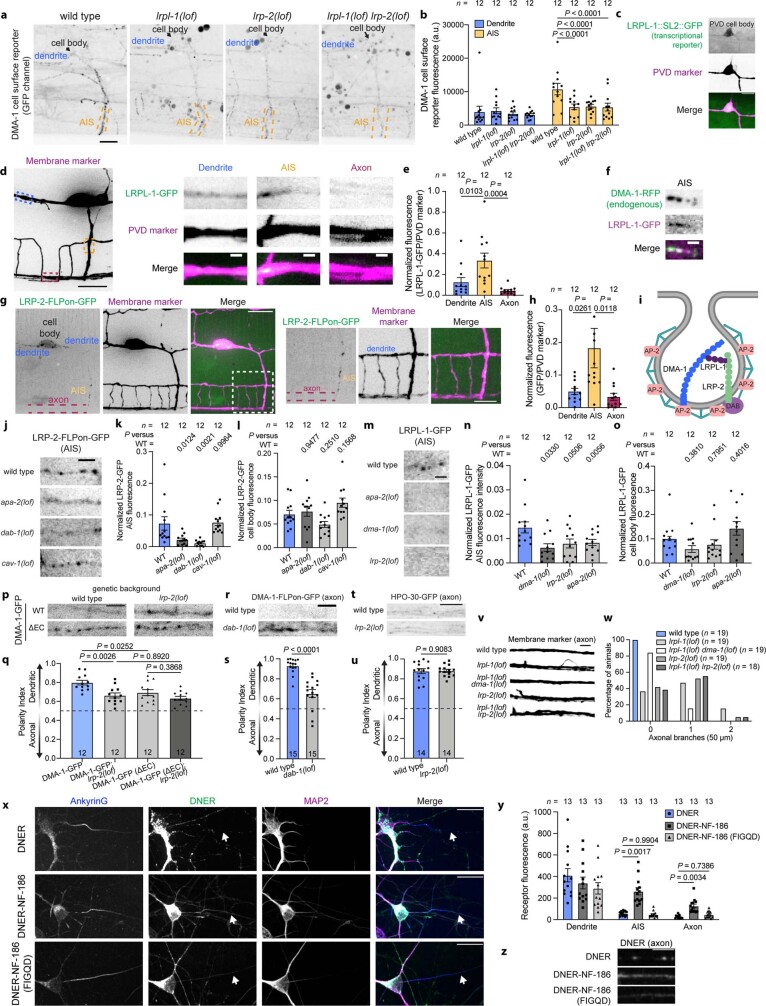
Molecular dissection and manipulation of AIS endocytic mechanisms. (**a**) GFP channel of the DMA-1 cell surface reporter in wild type and LRP protein mutant animals. (**b**) Quantification of GFP fluorescence from animals described in a. Wild type data is from Fig. 2k. (**c**) An SL2-GFP fusion at the C-terminus of the LRPL-1 endogenous locus showing LRPL-1 transcription in PVD. (**d**) Cell-specific expression of LRPL-1-GFP in PVD. Maximum Z projection of a 15 micron stack of the PVD membrane marker (left). Maximum Z projection of 0.65 micron stacks of the outlined regions on the left image (right). (**e**) Cell-specific LRPL-1-GFP fluorescence in PVD. (**f**) Endogenous DMA-1-RFP colocalizes with LRPL-1-GFP puncta in the AIS. (**g**) Cell-specific endogenous labelling of LRP-2-FLPon-GFP in PVD. Zoomed in region (indicated by the white box) is shown on the right. (**h**) Average LRP-2 fluorescence in neuronal subregions. (**i**) Model depicting two pathways for DMA-1 endocytosis. (**j**) Cell-specific endogenous LRP-2-FLPon-GFP localization in the AIS requires AP-2/APA-2 and DAB-1 function but not CAV-1. Average LRP-2-FLPon-GFP fluorescence in the (**k**) AIS and (**l**) cell body. (**m**) LRPL-1-GFP punctate localization in the AIS is dependent upon *apa-2, dma-1*, and *lrp-2*. Average LRPL-1-GFP fluorescence in the (**n**) AIS and the (**o**) cell body. (**p**) Cell-specific localization of wild type DMA-1-GFP or DMA-1 lacking its extracellular domain (DMA-1(ΔEC)) in the PVD neuron axon of wild type and *lrp-2* loss of function mutant animals. (**q**) DMA-1-FLPon-GFP polarity index of animals described in **p**. (**r**) Cell-specific endogenous DMA-1-FLPon-GFP localization in the axon of *dab-1* loss of function mutant animals. (**s**) DMA-1-FLPon-GFP polarity index of animals described in **r**. (**t**) Axonal regions of wild type and *lrp-*2 loss of function mutant animals cell-specifically expressing HPO-30-GFP. (**u**) HPO-30-GFP polarity index of animals described in **t**. (**v**) PVD axon labelled with a myristoylated GFP membrane marker in animals of the indicated genotype. (**w**) Axonal branches within the 50 microns distal to the AIS in animals described in **v** (*n* = 19 animals/genotype, except *lrpl-1 lrp-2* for which *n* = 18 animals). (**x**) Chimera localization in induced human neurons fixed and stained at DIV14. (**y**) Chimera fluorescence in the specified neuronal region. (**z**) Chimera fluorescence in the axon of cells shown in **x**. Arrows designate the cropped region. Brightness was increased for better visibility, and all conditions were processed the same. Data are shown as mean ± s.e.m. *N*-values are indicated on bar graphs and represent the number of animals or cells. *P* values were calculated as follows: **b**, **e**, **h**, **k**, **l**, **n**, **o**, and **q** using a two-way ANOVA with Šidák multiple comparison test; **s** and **u** using an unpaired two-tailed *t* test; **y** using a two-way ANOVA with Tukey’s multiple comparison test. Scale bars, 20 µm (**x**), 10 µm (**d** left, **g** left), 5 µm (**a**, **c**, **g** right, **p**, **r**, **t**, **v**, **z**), 2 µm (**j**) 1 µm (**d** right, **f**, **m**). Source data

To investigate the contribution of AIS endocytosis in maintaining the compartmentalization of polarized receptors, we took a two-pronged approach to perturb endocytosis at the AIS. Both approaches provided evidence that AIS endocytosis contributes to neuronal polarity. First, by investigating the mechanism of DMA-1 endocytosis, we gained molecular access to disrupt DMA-1's AIS endocytosis through inhibition of an LRP protein-mediated endocytic module that is preferential to the AIS (Supplementary Note 1). Loss-of-function mutations in *lrp-2* caused a decrease in DMA-1 dendritic polarity and a loss of neuronal function (Fig. 3f,g,j). Second, we designed a more specific and independent method to preferentially antagonize polarized receptor endocytosis at the AIS that can be applied to other receptors. Interaction with ankyrinG is known to prevent the endocytosis of AIS-resident proteins^9,26^. One such AIS-resident protein, NF-186, is endocytosed elsewhere in the neuron, but its endocytosis is inhibited specifically at the AIS by binding ankyrinG through a known motif^9,26^ (FIGQY). We genetically altered the endogenous DMA-1 locus by replacing its cytoplasmic tail with the NF-186 cytoplasmic tail. This chimaeric DMA-1–NF-186 receptor was enriched at the AIS, consistent with inhibited endocytosis (Fig. 4a,b). This change in receptor localization was dependent on the ankyrinG interaction. Introduction of a validated point mutation to disrupt ankyrinG binding (FIGQD) reversed the behaviour of the chimaeric receptor: it became punctate in the AIS and was not enriched there (Fig. 4a,b). We then used this chimaeric receptor strategy to examine polarity defects. We found that the DMA-1–NF-186 chimaera entered the axon, caused aberrant axonal branching, and elicited behavioural deficits in a harsh touch escape behavioural assay (Fig. 4c–f). Restoring DMA-1–NF-186 endocytosis at the AIS by introducing a single point mutation in the NF-186 ankyrinG-binding motif^9,25^ reversed these localization, morphological and functional defects (Fig. 4c–f). We then performed parallel experiments in induced human neurons by creating chimaeric fusion proteins with the delta/notch-like EGF repeat-containing receptor (DNER), a dendritically polarized single-pass type I transmembrane protein^45^. DNER–NF-186 also exhibited increased fluorescence at the AIS that is dependent on ankyrinG interaction (Extended Data Fig. 6x,y). We then examined polarity defects and found that DNER–NF-186 fluorescence was increased in the axon compared with DNER–NF-186 (FIGQD) (Extended Data Fig. 6x–z), consistent with a role of AIS endocytosis in receptor compartmentalization.

**Extended Data Fig. 7:**
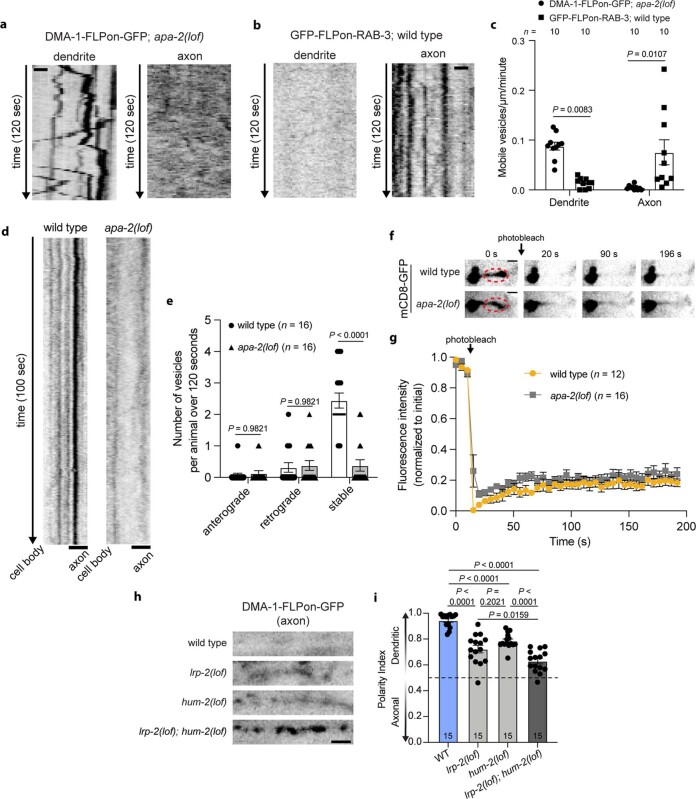
AIS endocytosis works in conjunction with known polarity mechanisms in the AIS to maintain neuronal polarity. (**a**) Time-lapse imaging of cell-specific endogenous DMA-1-FLPon-GFP in *apa-2* loss of function mutants shows robust vesicular trafficking in the dendrite but not axon. (**b**) Time-lapse imaging of cell-specific endogenous GFP-FLPon-RAB-3 shows robust vesicular localization and movement in the axon but not dendrite. (**c**) Quantification of vesicle dynamics described in **a**, **b**. (**d**) Kymograph analysis from time-lapse imaging of DMA-1-FLPon-GFP vesicles in the AIS of wild type and *apa-2(lof)* mutant animals. (**e**) Quantification of DMA-1-FLPon-GFP vesicular trafficking in the proximal AIS of wild type and *apa-2(lof)* endocytic mutant animals. (**f**) FRAP of mCD8-GFP in the AIS of wild type and *apa-2(lof)* endocytic mutants. *P* *=* 0.5842 using a two-way ANOVA to compare the two genotypes. (**g**) Fluorescence intensity of the red circled regions in **f**. Wild type data are from Extended Data Fig. 1i. (**h**) Cell-specific endogenous DMA-1-FLPon-GFP localization to the axon in the indicated animals. (**i**) DMA-1-FLPon-GFP polarity index of animals described in **h**. All data are shown as mean ± s.e.m. *N*-values are indicated on bar graphs and represent the number of individual animals scored for each condition.* P* values in **c** and **e** were calculated using a two-way ANOVA with Šidák multiple comparison test. *P* values in **i** were calculated using a one-way ANOVA with Šidák multiple comparison test. Scale bar, 2 µm (**d**, **f**, **h**), 1 µm (**a**, **b**). Source data

These data suggest that when dendritically polarized receptors are not removed from the AIS plasma membrane through endocytosis, they enter the axon, which diminishes their dendritic polarity. To understand how dendritic receptors enter the axon when endocytosis is inhibited, we used time-lapse imaging of DMA-1 to monitor vesicle entry into the axon of the *C. elegans* PVD neuron. When endocytosis was inhibited in *C. elegans* animals through a loss-of-function *apa-2* mutation, we did not observe robust DMA-1 vesicular trafficking in the axon (Extended Data Fig. 7a and Supplementary Videos 5 and 6), despite observing vesicle movement of axonally polarized GFP–FLPon–RAB-3 in the axon of wild-type *C. elegans* animals under the same imaging conditions (Extended Data Fig. 7b,c). Further, *apa-2* loss-of-function mutation had no effect on the known intracellular vesicle filter or membrane diffusion-barrier functions of the AIS (Extended Data Fig. 7d–g). Together, these results suggest that DMA-1 mislocalizes to the axon when endocytosis is inhibited through lateral diffusion in the membrane and not via intracellular vesicular trafficking pathways.

We then examined whether DMA-1 endocytic clearance from the AIS is a distinct mechanism that works in conjunction with known AIS polarity mechanisms. In principle, intracellular vesicle sorting and endocytic clearance from the plasma membrane could both contribute to neuronal polarity, as they dispatch distinct populations of proteins. To test this, we focused on myosin Va (also known as HUM-2 in *C. elegans*), which mediates dendritic protein intracellular vesicular trafficking in the AIS^14,29,30^, and LRP-2, which promotes AIS endocytosis of DMA-1 (Supplementary Note 1). Using double mutational analysis, we found that *lrp-2* and *hum-2* double-mutant *C. elegans* animals exhibit a further loss of DMA-1 dendritic polarity compared with single mutants, suggesting that these proteins function in separate pathways (Extended Data Fig. 7h,i). These results suggest that AIS endocytosis works in conjunction with the vesicle-filter function of the AIS for the parallel sorting of plasma membrane and intracellular receptors to maintain neuronal polarity.

**Extended Data Fig. 8:**
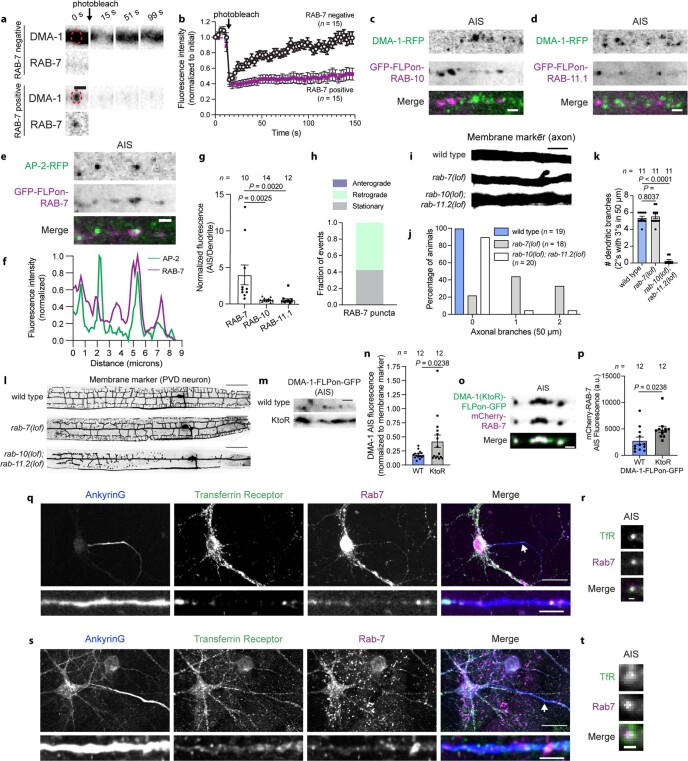
Dendritically polarized receptors are targeted to Rab7-positive late endosomes in the AIS. (**a**) DMA-1-GFP FRAP dynamics. (**b**) Quantification of FRAP experiments described in a. Endogenously labelled DMA-1-RFP and (**c**) GFP-FLPon-RAB-10 or (**d**) GFP-FLPon-RAB-11.1 in the AIS. (**e**) AP-2-RFP and endogenous GFP-FLPon-RAB-7 puncta rarely colocalize in the AIS. (**f**) Linescan analysis of fluorescence intensity of images in **e**. (**g**) Endogenous RAB protein average fluorescence. (**h**) Endogenous GFP-FLPon-RAB-7 vesicle dynamics in the AIS (*n* = 19 vesicles from 15 animals). (**i**) PVD axon labeled with a myristoylated GFP membrane marker in the indicated animals. (**j**) Axonal branches in the 50 µm distal to the AIS in animals described in **i**. (**k**) Number of secondary dendritic branches with tertiary branches in the proximal 50 µm of the anterior dendrite. (**l**) PVD neuron labelled with a myristoylated GFP marker in the indicated animals. (**m**) AIS localization of cell-specific endogenous DMA-1-FLPon-GFP with lysine to arginine mutations in the cytoplasmic tail (KtoR: K536R, K583R, K595R). (**n**) Endogenous DMA-1 fluorescence of animals described in **m**. (**o**) Cell-specific endogenous DMA-1-FLPon-GFP (KtoR) colocalizes with mCherry-RAB-7 in the AIS. (**p**) Endogenous DMA-1-FLPon-GFP (KtoR) increases mCherry-RAB-7 fluorescence in the AIS. (**q**) Confocal images of cultured mouse neurons fixed and stained at DIV14 for the indicated endogenous proteins. Bottom shows AIS zoom, and brightness is increased to improve visibility. (**r**) Zoomed in region of a Rab7-labelled vesicle in the AIS from the image shown in **q** (arrow designates the cropped region). Brightness is increased for better visibility. (**s)** Confocal images of human neurons fixed and stained at DIV26 for the indicated endogenous proteins. Bottom shows AIS zoom. Brightness is increased for better visibility. Extra-neuronal Rab7 fluorescence is from co-cultured glia. (**t**) Rab7-labelled vesicle containing TfR in the AIS from the image in **s** (arrow designates the cropped region). Brightness is increased for better visibility. Data are shown as mean ± s.e.m. *N*-values are indicated on bar graphs and represent the number of animals or cells. *P* values in **g** and** k** were calculated using a one-way ANOVA with Šidák multiple comparison test. *P* values in **n** and **p** were calculated using a two-tailed unpaired *t* test. Scale bars, 50 µm (**l**), 20 µm (**q** top, **s** top), 5 µm (**i**, **q** bottom, **s** bottom), 1 µm (**a**, **c**, **d**, **e**, **m**, **o**, **r**, **t**). Source data

For endocytosis in the AIS to prevent dendritic protein entry into the axon, endocytosed proteins must be either degraded or recycled back to the dendrite. Several lines of evidence support degradation. In the AIS, DMA-1 fluorescence overlapped with internal RAB-7-positive late endosomes but not RAB-10- and RAB-11-positive recycling endosomes (Fig. 4g,h and Extended Data Fig. 8a–d). RAB-7-positive late endosomes were a distinct population from AP-2-positive endocytic structures and were preferentially enriched in the AIS compared with RAB-10- and RAB-11-positive recycling endosomes (Extended Data Fig. 8e–g). We did not observe a pool of anterograde RAB-7-positive late endosomes in the AIS (Extended Data Fig. 8h), which suggests that DMA-1 is sorted to RAB-7-positive late endosomes for degradation after its endocytosis at the AIS. Consistent with a role for RAB-7, we found that *rab-7* mutation, but not *rab-10 rab-11* double mutation, caused aberrant axonal branching that coincides with a loss of DMA-1 dendritic polarity (Extended Data Fig. 8i,j). Conversely, *rab-10 rab-11* double mutation, but not *rab-7* mutation, resulted in a significant decrease in dendritic branching (Extended Data Fig. 8k,l). These results demonstrate distinct roles of Rab-mediated pathways in PVD neuronal morphology and suggest that post-endocytic trafficking of DMA-1 differs between the AIS and the dendrite. A role for RAB-7 implicates degradation through lysosomal pathways, which often involves ubiquitin-dependent sorting of transmembrane proteins for degradation. We next tested whether DMA-1 ubiquitination is required by endogenously mutating the three lysine residues in the cytoplasmic tail of DMA-1. Indeed, lysine to arginine mutations (DMA-1(KtoR)) caused a significant accumulation of DMA-1 in larger puncta in the AIS that colocalized with RAB-7-positive vesicles (Extended Data Fig. 8m,p). We also found that the dendritically polarized TfR partially colocalized with Rab7-positive late endosomes in the AIS of cultured mouse and human neurons (Extended Data Fig. 8q–t). The requirement for Rab7 and localization to the AIS make this endocytic clearance mechanism molecularly and functionally distinct from known trafficking modalities such as transcytosis, that function in polarity^46^.

## Generality of endocytosis in the AIS

**Extended Data Fig. 9:**
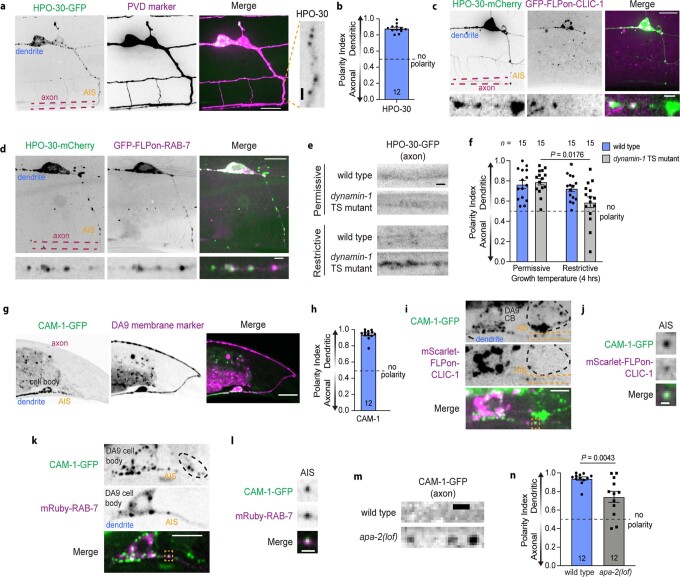
Diverse polarized receptors in *C. elegans* neurons are endocytosed in the AIS and require endocytosis for their polarity. (**a**) HPO-30-GFP localization in the PVD neuron. HPO-30-GFP is punctate in the AIS (inset). (**b**) HPO-30-GFP polarity index of animals described in a. (**c**) HPO-30-mCherry overlaps with CLIC-1-FLPon-GFP in the PVD neuron AIS. (**d**) HPO-30-mCherry overlaps with GFP-FLPon-RAB-7 in the PVD neuron AIS. (**e**) HPO-30-GFP in the axon of wild type or *dynamin-1* temperature sensitive (TS) mutants grown at the permissive (20 °C) or restrictive temperatures (32 °C, 4 h). (**f**) HPO-30-GFP dendritic polarity of animals described in **e**. (**g**) CAM-1-GFP localization in the DA9 neuron. (**h**) CAM-1-GFP polarity index of animals described in **g**. (**i**) CAM-1-GFP colocalizes with mScarlet-FLPon-CLIC-1 in the DA9 neuron AIS (left). Outlined in black is a neighbouring cell body. (**j**) Clathrin-coated vesicle containing CAM-1-GFP in the AIS of the animal shown in **i**. Yellow box indicates cropped region. (**k**) CAM-1-GFP colocalizes with mRuby-RAB-7 in the DA9 neuron AIS. Outlined in black is a neighbouring cell body. (**l)** RAB-7 labelled vesicle containing CAM-1-GFP in the AIS of animal shown in **k**. Yellow box indicates cropped region. (**m**) CAM-1-GFP mislocalizes to the axon in *apa-2* loss of function mutant animals. (**n**) CAM-1-GFP polarity index of animals described in **k**. Wild-type data are from Extended Data Fig. 9h. All data are shown as mean ± s.e.m. *N*-values are indicated on bar graphs and represent the number of individual animals. *P* values in **n** were calculated using a two-tailed unpaired *t* test. *P* values in **f** were calculated using a two-way ANOVA with Šidák multiple comparison test. Scale bars, 10 µm (**a**, **c** top, **d** top, **g**), 5 µm (**i**, **k**), 2 µm (**e**), 1 µm (**a** inset, **c** bottom, **d** bottom, **j**, **l**, **m**). Source data

**Extended Data Fig. 10:**
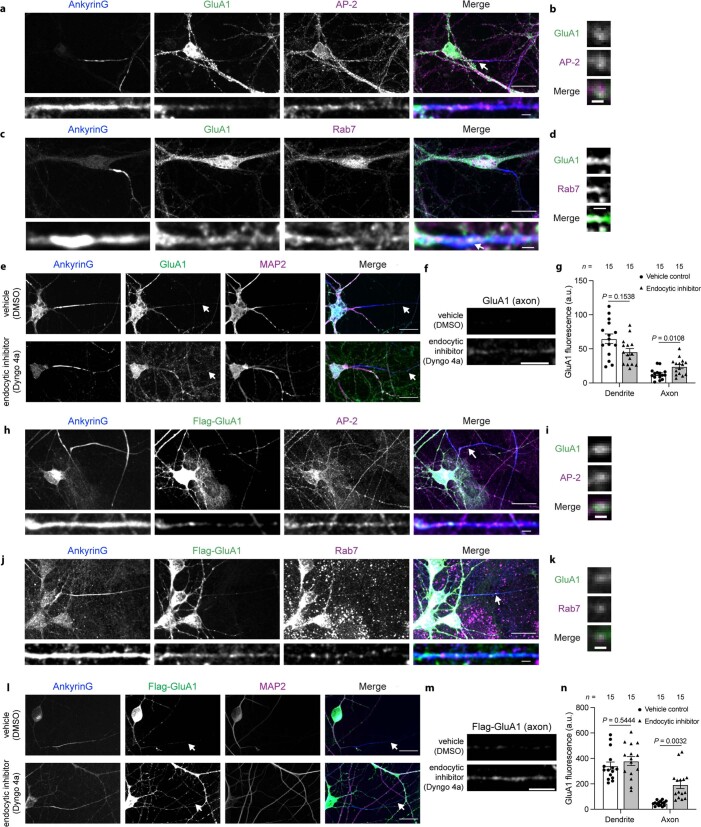
Dendritically polarized GluA1 receptors are endocytosed in the AIS and targeted to Rab7 positive late endosomes. (**a**) Confocal images of mouse neurons fixed and stained at DIV14 for the indicated endogenous proteins. Bottom shows AIS zoom (brightness is increased for better visibility). (**b**) AP-2 labelled vesicle containing GluA1 in the AIS from the neuron shown in a (arrow designates cropped region). Brightness is increased for better visibility. (**c**) Confocal images of mouse neurons fixed and stained at DIV14 for the indicated endogenous proteins. Bottom shows AIS zoom. (**d**) Rab7 labelled vesicle containing GluA1 in the AIS from the neuron shown in **c** (arrow designates cropped region). (**e**) Confocal images of mouse neurons pretreated with vehicle control (top, DMSO) or endocytic inhibitor (bottom, Dyngo 4a) for 5 h prior to being fixed and stained at DIV14. Arrows designate the proximal axon. (**f**) Proximal axonal region of neurons described in **e** (arrows show location of cropped region). (**g**) Average GluA1 fluorescence in the dendrite or proximal axon. (**h**) Confocal images of human neurons fixed and stained at DIV26 for the indicated proteins. Bottom shows AIS zoom (brightness is increased for better visibility). (**i**) AP-2 labelled vesicle containing Flag-GluA1 in the AIS from the neuron shown in **h** (arrow designates cropped region). Brightness is increased for better visibility. (**j**) Confocal images of human neurons fixed and stained at DIV26 for the indicated proteins. Bottom shows AIS zoom (brightness is increased for better visibility). (**k**) Rab7 labelled vesicle containing Flag-GluA1 in the AIS from the neuron shown in** j** (arrow designates cropped region). Brightness is increased for better visibility and extra-neuronal Rab7 fluorescence signal is from co-cultured glial cells. (**l**) Confocal images of human neurons pretreated with vehicle control (top, DMSO) or endocytic inhibitor (bottom, Dyngo 4a) for 18 h prior to being fixed and stained at DIV26. Arrows designate the axon. (**m**) Axonal region of neuron in l (arrows designate cropped region). (**n**) Average Flag-GluA1 fluorescence in the dendrite or axon. Data are shown as mean ± s.e.m. *N*-values are indicated on bar graphs and represent the number of cells. *P* values in **g** and **n** were calculated using a two-way ANOVA with Šidák multiple comparison test. Scale bars, 20 µm (**a** top, **c** top, **e**, **h** top, **j** top, **l**), 5 µm (**f**, **m**), 2 µm (**a** bottom, **c** bottom, **d**, **h** bottom,** j** bottom), 1 µm (**b**, **i**, **k**). Source data

Finally, we tested whether endocytic clearance of polarized receptors from the AIS is broadly used. We focused on an axonally polarized receptor and diverse dendritic receptors across multiple neuron types and species. We found that SER-1, a serotonin G protein-coupled receptor, was axonally polarized in the PVD neuron and enriched in AP-2-labelled clathrin-coated structures and RAB-7-positive vesicles in the AIS of *C. elegans* animals (Fig. 4i–k). These data support the notion that axonally polarized receptors are removed from the AIS membrane through endocytosis after initial intracellular vesicle trafficking to the axon distal to the AIS. Inhibition of SER-1 endocytosis through *apa-2* mutation caused a loss of axonal polarity and mislocalization to the dendrite (Fig. 4l,m). We found that the dendritically polarized claudin-like transmembrane protein, HPO-30, localized to clathrin-coated structures and RAB-7-positive vesicles in the AIS, and endocytic inhibition caused a loss of its dendritic polarity in the *C. elegans* PVD neuron (Extended Data Fig. 9a–f). In the *C. elegans* DA9 neuron, the dendritically polarized ROR1/2 tyrosine kinase receptor, CAM-1, localized to clathrin-coated structures and RAB-7-positive vesicles in the AIS, and endocytic inhibition decreased its dendritic polarity (Extended Data Fig. 9g–n). In cultured mouse and induced human neurons, we found that the dendritically polarized glutamate receptor^47^, GluA1, partially localized to both AP-2-labelled endocytic structures and Rab7-positive vesicles in the AIS, and endocytic inhibition diminished its dendritic compartmentalization (Extended Data Fig. 10a–n). Therefore, we find that diverse polarized receptors in multiple neuron types and species are endocytosed in the AIS to be removed from the AIS plasma membrane.

## Discussion

We propose a mechanism in which axonally and dendritically polarized transmembrane proteins enter the AIS through lateral diffusion, are captured for endocytosis, internalized from the plasma membrane, and targeted for degradation (Fig. 4n). Numerous polarized receptors partially localize to clathrin-coated structures and Rab7-positive late endosomes in the AIS across multiple neuron types and species. Although we find that endocytosis in the AIS is coupled to degradation, both DMA-1 and TfR require recycling in the dendrite^48,49^. This suggests that the post-endocytic fate of receptors differs between the AIS and dendrite. Endocytosis and subsequent degradation of polarized receptors inherently functions as a clearance mechanism, and thus may be critical for reinforcing compartment boundaries in the AIS and maintaining neuronal polarity. Through molecular manipulations of DMA-1, we find that preferentially perturbing AIS endocytosis results in a loss of its dendritic compartmentalization. Our analysis with the DNER receptor suggests this may represent a general mechanism used to maintain axon–dendrite compartmentalization.

Endocytic clearance of polarized receptors from the AIS membrane is likely to work in concert with known polarity mechanisms. We found that AIS endocytosis functions in parallel with myosin Va mediated intracellular vesicle trafficking. The AIS diffusion barrier has been shown to trap proteins in the AIS between 190-nm-spaced periodic ring-like structures that comprise the actin–spectrin-based submembranous cytoskeleton^10,21^. We anticipate that the membrane region between these structures can accommodate endocytic vesicle formation and that endocytosis removes transmembrane proteins that are trapped by the diffusion barrier. The combination of polarized vesicular trafficking, the diffusion barrier and endocytic clearance in the AIS may enable the neuron to maintain polarity over its long lifespan.

Our results elucidate an endocytic clearance function of the AIS in neuronal polarity. Through the study of one of the most polarized cell types, the present results reveal a mechanism by which neurons can achieve strict compartmentalization even along a contiguous membrane. Whether other polarized cell types or cells with specialized functional membrane domains use such a mechanism to reinforce compartment boundaries on the membrane is subject to future investigation. AIS endocytosis, which is critical for neuronal polarity and neuronal function, is therefore likely to have widespread physiological significance.

## Methods

### *C. elegans* methods

*C. elegans* animals were cultured at 20 °C on NGM plates using OP50 *Escherichia coli* as a food source according to standard procedures unless otherwise noted^50^. N2 Bristol was used as the wild-type reference strain. *C. elegans* transgenic strains were prepared by microinjection of the construct of interest (using 1–10 ng µl^−1^ of plasmid DNA) and co-injected with selection marker plasmids *Podr-1::RFP* (100 ng µl^−1^), *Podr-1::GFP* (100 ng µl^−1^) or *Punc-122::BFP* (100 ng µl^−1^) to aid in selection of transgenic animals. Two constructs were used as PVD membrane markers: *ser2prom3::myristoylated::mCherry* when examining fluorescent protein localization and *ser2prom3::myristoylated::GFP* when examining neuron morphology. Detailed descriptions of *C. elegans* strains used in this study are provided in Supplementary Table 2. *C. elegans* hermaphrodites were analysed at the L4 larval stage unless otherwise indicated. Dynamin-1 inhibition was obtained by shifting *dynamin-1* temperature-sensitive mutant *C. elegans* animals to the restrictive temperatures (32 °C) for 4 h before imaging. Wild-type *C. elegans* animals were grown, treated and imaged in parallel.

### Constructs and cloning

All constructs are described in Supplementary Table 3. Constructs generated during this study for use in *C. elegans* experiments were created with an isothermal assembly method using overlapping oligonucleotides^51^. Flippase-mediated recombination was used to achieve selective labelling of endogenous fusion proteins in a limited number of cells^52^. *C. elegans* expression constructs were made in a pSM delta vector. *ser2prom3*::FLP was assembled from a 4,141-nucleotide 5′ promoter sequence of *ser-2* and a 2× nuclear localization sequence (NLS) FLP recombinase sequence from pMLS262^53^. *ser2prom3*::NF-186 (rat) and *ser2prom3*::NF-186(FIGQD) (rat) were assembled from a 4,141-nucleotide 5′ promoter sequence of *ser-2* and 3,516-nucleotide rat HA–NF-186 sequence^25^ (Addgene plasmid #31061). The FIGQD point mutation was introduced via the reverse cloning primer to amplify the NF-186 sequence: CCTTTACTCATAGAGCCGCTTCCACTACCcccgggACTTCCGCTGCCggccagggaatagatggcattgactggagatgtggcctctgagctctcattgccctcggtctcctccttgtcctttctgacagtgtCctggccaataaaggagccatcttc.

### *C. elegans* genome editing using CRISPR–Cas9

Endogenous fluorophore insertions, basepair changes or deletions and chimeric receptors were created by gonadal microinjection of CRISPR–Cas9 protein complexes. CRISPR–Cas9 genome editing was performed using standard protocols^54^. Cas9 protein and tracRNA (IDT) were both injected at 1.525 µM. crRNAs (IDT) were injected at a concentration of 1.525 µM. DNA repair templates were generated using PCR or ordered as ultramers (IDT) and injected at 0.15–3 µM. *dpy-10(cn64)* was used as a co-injection marker and outcrossed once the desired genome edit was generated. F_2_ *C. elegans* animals were screened for the desired genome edit using PCR and confirmed by sequencing. Guide RNAs used for genome editing are listed in Supplementary Table 4.

### *C. elegans* confocal microscopy imaging and analysis

Images of fluorescently tagged fusion proteins were captured at room temperature in live *C. elegans* animals. Mid-L4 stage hermaphrodite *C. elegans* animals were anaesthetized using 10 mM levamisole (Sigma-Aldrich) in M9 buffer and mounted on 5% agarose pads for imaging. *C. elegans* animals were imaged on either an inverted Zeiss Axio Observer Z1 microscope equipped with a Hamamatsu EM-CCD digital camera, a Yokogawa CSU-X1 spinning-disk unit, controlled by Metamorph (version 7.8.12.0), and using either a Plan-Apochromat 100× 1.4 NA objective or a 63× 1.4 NA objective or on an inverted Zeiss Axio Observer Z1 microscope equipped with a Yokogawa CSU-W1 spinning-disk unit, a Prime 95B Scientific CMOS camera, controlled by 3i Slidebook (v6), and using either a C-Apochromat 40× 0.9 NA or 63× 1.2 NA objective. FRAP experiments were performed on the above microscope using a Vector diffraction-limited laser scanner (3i). In all cases, image settings (for example, exposure time and laser power) were identical for all genotypes across the experiment.

Quantitative image analysis was performed on unprocessed images using Fiji Software^55,56^. Maximum-intensity projections were rotated, cropped, and straightened to generate display images. Brightness and contrast were adjusted in Fiji to show relevant features, treating images being compared in the same manner. Kymographs were generated using Fiji. Line scan analysis of AIS regions was performed on cropped AIS images using the plot profile function in Fiji. Manders’ overlap coefficient was calculated using the Coloc2 plugin in Fiji to quantify endogenous RAB fluorescence overlap with endogenous DMA-1 fluorescence in the AIS. Overlap was calculated by drawing a region of interest around the AIS and using Costes’ thresholding. Puncta density was calculated using fluorescence thresholding and automated particle counting to determine the number of puncta per micron using the standard thresholding and particle analysis functions in Fiji.

To calculate dendritic polarity index, the dendrite or axon of PVD was traced based on the general mCherry membrane marker using the freehand selection function in Fiji. The mean pixel intensity of the fluorescently tagged protein in the dendrite (DP) and axon (AP) and the membrane-bound mCherry in the dendrite (DM) and axon (AM) were measured separately. The normalized mean intensity of the fluorescence-tagged protein in the dendrite (*D*) or the axon (*A*) is calculated by: *D* = DP/DM and *A* = AP/AM. We normalized the GFP signal of the cargo to the membrane-bound mCherry because the left and right PVD axonal regions overlap, but the contributions of each neuron to membrane-bound mCherry and cargo GFP are proportional. The polarity index (PI) for each worm was obtained by: PI = *D*/(*A* + *D*). PI is 0 for a completely axonal localization, 1 for a completely dendritic localization, and 0.5 for a non-polarized localization. All dendritic polarity indices shown were calculated based on the proximal anterior dendrite region though no differences were found compared to the proximal posterior dendrite region.

### Harsh touch escape response assay

Harsh touch escape response assays were performed by touching day 1 adult *C. elegans* animals with a platinum wire pick in the midsection of the body as described previously^57^. The tip of the wire platinum pick was flattened and cut to be 20 mm thick and 30 mm wide. For each genotype, 11–12 *C. elegans* animals were tested 10 times with a 10 min interval between trials. All *C. elegans* animals used for this assay were in the *mec-4(e1611)* light touch mutant background to isolate the harsh touch response except for *mec-3(e1338)* animals that were used as a control^58^. *C. elegans* animals carrying mutations in *degt-1* and *mec-3*, which encode a DEG/ENaC channel and a homeobox transcription factor that controls the differentiation of touch receptor neurons, respectively, were included as control animals with known behavioural defects^58,59^. These experiments were performed and analysed blind to genotypes.

### Pulsed-pH protocol

The TfR-SEP plasmid was used^42^ and described^60^ previously. The mScarlet-Na_v_II-III plasmid was constructed as follows: the fragment corresponding to loop II–III of the Nav1.2 sodium channel was extracted from the YFP-Na_v_II-III plasmid (Addgene #26056) by PCR (forward: TAGTCGATCGCCAGTTCTTTCAGCTCAGACAACCTG; reverse CACTCGAGCTAGCTTATCTTGTAGCACGTT), digested with PvuI and XhoI and ligated into a pmScarlet_ABHD6 vector (gift from B. Fakler), which leads to the expression of mScarlet–Na_v_II–III under the control of a CMV promoter. The construct was verified by sequencing.

Pregnant Sprague-Dawley rats (Janvier Labs) were housed at 50–70% humidity and 18–22 °C with ad libitum feeding and a 12 h light-dark cycle. Neurons were cultured following the Banker method^61^ as described before^42^. All procedures were in accordance with the European guide for the care and use of laboratory animals and approved by the ethics committee of Bordeaux University (CE50) and the French Ministry of Research.

Neurons were transfected at 6 days in vitro (DIV) with TfR–SEP alone or together with mScarlet–Na_v_II–III by the calcium phosphate method. Live cell recordings were performed at 9–10 DIV. Neurons were perfused with HEPES buffered saline solution (HBS) at 37 °C. HBS contained (in mM): 120 NaCl, 2 KCl, 2 MgCl_2_, 2 CaCl_2_, 5 d-glucose and 10 HEPES, and was adjusted to pH 7.4 and 260–270 mOsm. For the pulsed-pH assay, MES buffered saline solution (MBS) was prepared similarly by replacing HEPES with MES and adjusting the pH to 5.5. All salts were from Sigma-Aldrich. The field of view was centred on the AIS as visualized either with mScarlet–NavII–III or endogenous neurofascin fluorescence. Endogenous neurofascin was visualized by labelling with anti-Neurofascin antibody diluted in HBS (1:250) (Monoclonal mouse (clone A12/18), Antibodies Incorporated 75-172, RRID AB_2282826) for 10 min and then labelled with anti-mouse Alexa568 (Thermo Fisher Scientific, A10037, RRID AB_2534013) in HBS (1:500) for 3 min. The field of view also contained part of the soma and some dendrites (17 out of 21 recordings for mScarlet–NavII–III expression and 12 out of 20 recording for Neurofascin labelling), which were used for comparison. HBS and MBS were perfused locally around the recorded cell using a 2-way borosilicate glass pipette as described previously^40^. Imaging was performed with an Olympus IX71 inverted microscope equipped for total internal reflection fluorescence microscopy with a 150×, 1.45 NA objective (UAPON150XOTIRF), a laser source (Cobolt Laser 06-DPL 473 nm, 100 mW) and an Ilas2 illuminator (Gataca Systems) with a penetration depth set to 100 nm. Emitted fluorescence was filtered with a dichroic mirror (R405/488/561/635) and an emission filter (ET525/50m, Chroma Technology) and recorded by an EM-CCD camera (QuantEM 512C, Princeton Instruments) controlled by Metamorph (version 7.10.3.279). Videos were acquired for 10 min at 0.5 Hz.

Detection of endocytic events and their analyses was conducted using previously described, custom-made MATLAB scripts^40,42^. In short, a sudden punctate fluorescence increase appearing in pH 5.5 images was detected as being an endocytic event if: (1) it was visible for more than 3 frames (that is, 8 s), and (2) it appeared at the same location as a pre-existing fluorescence cluster detectable in pH 7.4 images. Candidate events from each cell were validated using a trained support vector machine as described previously. Event frequency was expressed per cell surface area measured on the cell masks drawn around the AIS, a selected dendrite, or the entire neuron.

For immunocytochemistry, cells were fixed for 10 min in warm 4% paraformaldehyde/4% sucrose in phosphate buffered saline solution (PBS). After a rinse with PBS, cells were permeabilized with 0.1% Triton X-100 in PBS containing 1% gelatin (PGT buffer) (to block unspecific binding) for 20 min. After rinse, neurons were incubated in a mix of anti-Neurofascin antibody (1:500) and anti-mScarlet antibody (1:500) (Polyclonal chicken antibody, Synaptic Systems, 409006, RRID AB_2725776) diluted in PGT, followed by Alexa Fluor 647 conjugated anti-mouse antibody (1:500) (Thermo Fisher Scientific, A21235, RRID AB_2535804) and Alexa Fluor 594 conjugated anti-chicken antibody (1:500) (Thermo Fisher Scientific, A11042, RRID AB_2534099). The samples were mounted in Fluoromount-G with DAPI. The samples were imaged with Leica DM5000 epifluorescent microscope, with appropriate filters for fluorophore wavelengths and a sCMOS digital camera (Hamamatsu Flash 4.0 V2) controlled by Metamorph (version 7.8.4.0).

### Transferrin ligand uptake assay

For uptake of transferrin ligand, cultured rat neurons were starved for 10 min in HBS at 37 °C, and then incubated with transferrin–Alexa647 (Thermo Fischer Scientific, T23366,) at 50 μg ml^−1^ in HBS for 10 min at 37 °C. After a quick wash with cold HBS, cells were stripped of surface-bound transferrin–Alexa647 with glycine buffer (100 mM NaCl, 50 mM glycine, pH 3, 300 mOsm) twice, rinsed with cold HBS and fixed with 4% paraformaldehyde/4% sucrose in PBS at room temperature for 15 min. For surface labelling, neurons were incubated with transferrin–Alexa647 for 10 min at 4 °C, followed by a quick wash with cold HBS and fixed. The cells were then permeabilized with PGT and labelled with anti-TfR antibody (1:1,000) (clone H68.4, Thermo Fisher Scientific, 13-6800, RRID AB_2533029) followed by anti-mouse Alexa568 conjugate (1:500) (Thermo Fisher Scientific, A11004, RRID AB_2534072). The samples were mounted in Fluoromount-G with DAPI and were imaged on a spinning-disk confocal microscope with a 63× objective and 488, 561 and 634 nm illumination. A stack of focal planes, 0.5 μm apart, was acquired for the GFP, Alexa568 and Alexa647 channels. A maximum-intensity projection of all channels was used for quantification of fluorescence measurements. We defined a mask of the cell in the Alexa568 channel and used it for quantification of transferrin–Alexa647 labelling.

### Embryonic stem cell culture

Male human embryonic stem cell line H1 was cultured under feeder-free conditions in mTeSR1 medium (Stem Cell Technologies). In brief, medium was replaced every day. When 80–90% confluent, cells were treated with Accutase (Innovative Cell Technologies), collected and centrifuged at 1,000 rpm for 3 min, re-suspended in mTeSR1 with Thiazovivin (2 μM, Bio Vision), and plated onto Matrigel (BD Biosciences)-coated plates. Mouse glial cells were isolated from the forebrain of newborn wild-type CD1 (Charles River) mice. Mice were housed at room temperature (20–22 °C) with 30–70% humidity, and a 12 h light-dark cycle. In brief, newborn mouse forebrain was digested with papain for 30 min and plated onto 10 cm dishes in DMEM (Thermo Fisher) supplemented with 10% calf serum (GE healthcare life sciences), sodium pyruvate (Thermo Fisher), MEM Non-Essential Amino Acids (Thermo Fisher), penicillin/streptomycin (Thermo Fisher), and 2-mercaptoethanol (Sigma). All mouse procedures were approved by the administrative panel on laboratory animal care (APLAC), Stanford University.

### Lentiviral generation

Lentiviruses were produced in HEK293T cells (ATCC) as described previously^62^. The expression vector and three helper plasmids (pRSV–REV, pMDLg/pRRE and VSV-G) were co-transfected with polyethylenimine. Lentiviral particles were ultra-centrifuged, re-suspended in DMEM, snap-frozen, and stored at −80 °C. The following lentivirus constructs were used: FUW-TetO-Ngn2-T2A-puromycin, FUW-TetO-Ngn2-T2A-blasticidin, FUW-TetO-Flag-GluA1-T2A-puromycin, FUW-TetO-Flag-DNER-T2A-puromycin, FUW-TetO-Flag-DNER-NF-186-T2A-puromycin, FUW-TetO-Flag-DNER-NF-186(FIGQD)-T2A-puromycin and FUW-rtTA. FUW-TetO-Flag-DNER plasmids were subcloned from Addgene plasmid #51053 and the FUW-TetO-Flag-GluA1 plasmids were subcloned from Addgene plasmid #64942 using Gibson assembly methods.

### Generation of induced neuron cells

Ngn2-iN cells were generated as described^63^. Embryonic stem cells were plated as dissociated cells in 6 cm dish (∼8 × 10^5^ cells) on day 0. At the time of plating, cells were infected with lentiviruses containing expression constructs of Ngn2-T2A-puromycin or Ngn2-T2A-blasticidin and rtTA. On day 1, the culture medium was replaced with N3 medium (DMEM/F12 (Thermo Fisher), N2 (Thermo Fisher), and MEM Non-Essential Amino Acids (Thermo Fisher) supplemented with insulin (Sigma)) containing doxycycline (2 μg ml^−1^, Sigma) to induce gene expression, and the culture was retained in the doxycycline containing-medium for ∼2 weeks. From day 2 to day 4, puromycin or blasticidin was used to select transduced cells. On day 3, lentivirus expressing Flag-labelled receptors were added to the plate for experiments requiring expression of Flag-labelled receptors. On day 5, AraC (4 μM, Sigma) was added to remove dividing cells. In cells treated with FUW-TetO-Flag-GluA1-T2A-puromycin, FUW-TetO-Flag-DNER-T2A-puromycin, FUW-TetO-Flag-DNER-NF-186-T2A-puromycin, or FUW-TetO-Flag-DNER-NF-186(FIGQD)-T2A-puromycin lentivirus, puromycin was added for selection. On day 6, iN cells were dissociated by Accutase, centrifuged as above, re-suspended in Neurobasal (Thermo Fisher) with B27 (Thermo Fisher), Glutamax (Thermo Fisher), and 5% fetal bovine serum (FBS, GE Healthcare). Passage 3 mouse glial cells were added to cell suspension and plated on matrigel-coated coverslips (5–7.5 × 10^4^ iN cells and 3 × 10^4^ mouse glial cells per well of a 24-well plate). On day 8, medium was changed to Neurobasal with B27, Glutamax, 2% FBS, and AraC (4 μM). Thirty per cent of the medium was exchanged every three to four days. Cultures were analysed at two and four weeks after induction of the transgenes.

### Primary mouse hippocampal cultures

C57BL/6J mice (Jackson 000664) were housed at room temperature and 40–60% humidity with a 12 h light-dark cycle with free access to food and water ad libitum. Hippocampi were dissected from P0 neonatal male and female wild-type mice, dissociated by papain digestion, filtered through a 70-µm cell strainer and plated on poly-d-lysine coated coverslips in 24-well plates. Cultures were maintained in Neurobasal A medium supplemented with l-glutamine, B27 and AraC in humidified incubator with 5% CO_2_ at 37 °C. The neurons were cultured for 14 days before experiments. All mouse procedures were approved by the administrative panel on laboratory animal care (APLAC), Stanford University.

### Immunostaining of cultured mouse and induced human neurons

DIV14 mouse neuron cultures were washed with PBS 3 times for 1 min. Cells were then fixed with 4% paraformaldehyde in PBS at room temperature for 15 min. Cells were washed 3 times for 5 min and then incubated in blocking and permeabilization buffer for 1 h at room temperature (5% normal donkey serum, 0.3% Triton-100, 0.05% sodium azide in PBS). Primary antibodies diluted in TBST (0.1% tween in TBS) were added and incubated overnight at 4 °C. Cells were then washed 3 times with TBST for 5 min before incubation with secondary antibodies diluted in TBST for 1 h at room temperature. Cells were washed 3 times for 10 min with TBST and then mounted with Prolong Diamond Anti-fade Mountant (Thermo) on glass slides for imaging. In experiments with Dyngo 4a treatment, 30 μM Dyngo 4a was used in serum-starved conditions with DMSO used as a vehicle control.

DIV14 or DIV26 induced human neuron cultures were washed with PBS+++ buffer (0.5 mM CaCl_2_, 1 mM MgCl_2_, 4% sucrose in PBS). Cells were then fixed with PFA+ buffer (4% paraformaldehyde and 4% sucrose in PBS) for 10 min at room temperature. Cells were then washed 3 times with PBS. Cells were permeabilized with 0.2% Triton X in PBS for 10 min and washed 3 times with PBS. Cells were then incubated in blocking buffer (4% BSA, 1% cosmic calf serum in PBS) for one hour at room temperature. Primary antibodies diluted in blocking buffer were added and incubated overnight at 4 °C. Cells were washed 4 times with PBS before adding secondary antibodies diluted 1:1,000 in blocking buffer for 1 h at room temperature. Cells were washed 3 times in PBS and then mounted with DAPI Fluoromount-G (Southern Biotech) or Prolong Diamond Anti-fade Mountant (Thermo) on glass slides for imaging. In experiments with Dyngo 4a treatment, 30 μM Dyngo 4a was used in serum-starved conditions with DMSO used as a vehicle control.

The following primary antibodies were used for immunofluorescence studies: MAP2 (Abcam ab5392 (1:1,000), Abcam ab32454 (1:500)); MAP2A/B (EnCor Biotech GPCA-MAP2A/B (1:500)); ankyrinG (clone N106/36, Sigma MABN466 (1:1,000), Synaptic Systems 386 003 (1:500), Synaptic Systems 386 005 (1:500)); clathrin heavy chain (Abcam ab21679 (1:500)); AP-2 complex subunit α1 (Abcam ab189995 (1:500)); α-adaptin (clone AP6, Thermo MA1-064 (1:500)); RAB-7 (EPR7589, Abcam ab137029 (1:250)); RAB7A/RAB7 (LSBio LS-B13237-100 (1:250)), TGN46 (BioRad AHP500G (1:1,000)); βIII tubulin (Abcam ab41489 (1:1,000)); L1CAM (clone UJ127.11, Sigma L4543 (1:100)); glutamate receptor 1 (Sigma AB1504 (1:100)) and Flag (Millipore Sigma F7425 (1:1,000)). All antibodies used are standard commercial antibodies. All staining was done post-fixation except for L1CAM. To label L1CAM in human neurons, a live staining was performed. The human-specific clone UJ127.11 (Sigma-Aldrich) was added to fresh culturing medium for 30 min at 37 °C (20 μg ml^−1^) before washing and fixation as described above.

The following secondary antibodies were used for immunofluorescence studies (all diluted 1:500): rabbit polyclonal anti-Goat IgG (H+L) Alexa Fluor 488 (Thermo Fisher A27012), donkey polyclonal anti-mouse IgG (H+L) Alexa Fluor Plus 488 (Thermo Fisher A32766), goat polyclonal anti-chicken IgY (H+L) Alexa Fluor 555 (Thermo Fisher A21437), donkey polyclonal anti-goat IgG (H+L) Alexa Fluor Plus 555 (Thermo Fisher A32816), goat polyclonal anti-rabbit IgG (H+L) Alexa Fluor 555 (Thermo Fisher A27039), donkey polyclonal anti-sheep IgG (H+L) Alexa Fluor 555 (Thermo Fisher A21436), goat polyclonal anti-chicken IgY (H+L) Alexa Fluor 647 (Thermo Fisher A21449), goat polyclonal anti-rabbit IgG (H+L) Alexa Fluor 647 (Thermo Fisher A32733), DyLight 405 AffiniPure Donkey Anti-Chicken IgY (IgG) (H+L) (Jackson 703-475-155), DyLight 405 AffiniPure Donkey Anti-Mouse IgG (H+L) (Jackson 715-475-151), Alexa Fluor 488 AffiniPure Donkey Anti-Rabbit IgG (H+L) (Jackson 711-545-152), Alexa Fluor 488 AffiniPure Donkey Anti-Goat IgG (H+L) (Jackson 705-545-147), Alexa Fluor 594 AffiniPure Donkey Anti-Rabbit IgG (H+L) (Jackson 711-585-152), Alexa Fluor 647 AffiniPure Donkey Anti-Mouse IgG (H+L) (Jackson 715-605-151), Alexa Fluor 647 AffiniPure Donkey Anti-Chicken IgY (IgG) (H+L) (Jackson 703-605-155), Alexa Fluor 647 AffiniPure Donkey Anti-Guinea Pig IgG (H+L) (Jackson 706-605-148) and Donkey anti-Goat IgG (H+L) Highly Cross-Adsorbed Secondary Antibody Alexa Fluor Plus 647 (Thermo A32849).

Images were rotated, cropped, and straightened to generate display images. Images were analysed by measuring the mean fluorescence intensity in the indicated region and background subtracted using a nearby region of the coverslip without cells. Only cell regions without overlapping neurites were analysed. Neurons were selected for analysis using the following criteria: (1) having a clearly identifiable cell body with an AIS that projects away from the cell body (based on strong ankyrinG labelling) and (2) having an AIS that was easily followed to the axonal region. Axonal regions were selected as being distal to the strong ankyrinG labelling of the AIS. These axonal regions also had markedly lower MAP2 staining compared to the dendritic domains of the neuron.

### Interaction screening and protein biochemistry

The interaction screening was based on the modified extracellular interactome assay strategy as reported previously^64^ and adapted to 384-well plates to accommodate the larger number of proteins being screened. 380 proteins chosen from common neuronal cell-surface receptor families were cloned into *Drosophila* culture expression plasmids in bait format with an Fc tag and in prey format with a pentameric coiled coil domain followed by the human placental alkaline phosphatase. All proteins were expressed and secreted using *Drosophila* S2 cells following transient transfection with the TransIT-Insect transfection reagent (Mirus, MIR 6104) according to the manufacturer’s directions without modification. Bait proteins were captured directly from conditioned medium onto Protein A-coated 384-well plates overnight at 4 °C, washed with 1× PBS including 0.1% BSA, 1 mM MgCl_2_ and 1 mM CaCl_2_, followed by a 3-h incubation at room temperature by the conditioned medium for prey proteins, and a second wash. Binding was detected using the absorbance from the chromogenic substrate KPL BluePhos (Seracare, 5120-006) at 650 nm. For analysing results, we followed the trimmed *Z*-score strategy as previously implemented^65^, where the lowest and highest values (10% each) of measured absorbances were removed to calculate trimmed mean and standard deviations for each collection of measurements, which were then used to calculate *Z*-scores (that is, s.d. over mean) for all measurements.

For the surface plasmon resonance experiment, DMA-1 ectodomain (amino acids Leu20 to Leu507) was cloned in a modified baculoviral transfer plasmid based on pAcGP67A, designed to secrete proteins with C-terminal Avi tag for biotinylation and hexahistidine tag for protein purification. Similarly, LRPL-1 cDNA lacking the signal peptide was cloned into pAcGP67A with a hexahistidine tag. Both proteins were successfully secreted into medium from High Five cells upon infection with baculoviruses, demonstrating that LRPL-1 is a secreted protein. Proteins were purified with immobilized metal affinity chromatography with Ni-NTA Agarose resin (Qiagen), followed by size-exclusion chromatography in HBS (10 mM HEPES pH 7.2, 150 mM NaCl). LRPL-1 was incubated with the *E. coli* biotin ligase BirA for biotinylation. SPR was performed using a Streptavidin chip on a Biacore X100 (GE Healthcare) for capturing LRPL-1 in HBS with 0.05% Tween-20 and 0.1% Bovine serum albumin to decrease non-specific binding of DMA-1 to the chip surface. The sensorgrams were analysed with the manufacturer’s evaluation software (Biacore X100 Evaluation software, version 2.0.2) using kinetic and equilibrium models of binding. The kinetic fits yielded on and off rates (*k*_on_ and *k*_off_ values) of 4.6 × 10^3^ M^−1^s^−1^ and 0.018 s^−1^, respectively, resulting in a dissociation constant (*K*_D_) of 3.9 µM. The steady-state analysis gave a similar *K*_D_ of 6.9 µM. Both analyses suffered from limited non-specific binding, which likely explains the two-fold discrepancy between the two *K*_D_ values.

### Statistics and reproducibility

All data are expressed as the mean ± s.e.m. Sample size refers to the number of *C. elegans* animals or neurons. Scatter plots show an overlay of mean and s.e.m., and each dot represents an individual *C. elegans* animal, neurons or neuronal region. Therefore, *n* is the number of dots in each scatter plot. Statistical comparisons were performed in Prism 8.0 software (GraphPad Software). Statistical significance tests are specified in the figure legends. Each *C. elegans* in vivo measurement (FRAP or fluorescence intensity) is from a distinct animal. All *C. elegans* in vivo imaging data were replicated in at least two independent imaging sessions. Each vertebrate neuron measurement is from a distinct neuron. All vertebrate neuron imaging data were from at least three independent neuronal cultures and at least three independent imaging experiment sessions. All experiments showing representative data were repeated with similar results. Independent experiments represent independent biological replicates.

### Reporting summary

Further information on research design is available in the Nature Research Reporting Summary linked to this article.

## Online content

Any methods, additional references, Nature Research reporting summaries, source data, extended data, supplementary information, acknowledgements, peer review information; details of author contributions and competing interests; and statements of data and code availability are available at 10.1038/s41586-022-05074-5.

## Supplementary information

Supplementary Information This file contains a Supplementary Note, Supplementary Fig. 1, legends for Supplementary Tables 1–4 and Supplementary Videos 1–6, and Supplementary References

Reporting Summary

Supplementary Table 1

Supplementary Table 2

Supplementary Table 3

Supplementary Table 4

Supplementary Video 1

Supplementary Video 2

Supplementary Video 3

Supplementary Video 4

Supplementary Video 5

Supplementary Video 6

## Source data

Source Data Fig. 1–4 and Extended Data Fig. 1–10

## Figures and Tables

**Extended Data Fig. 5:**
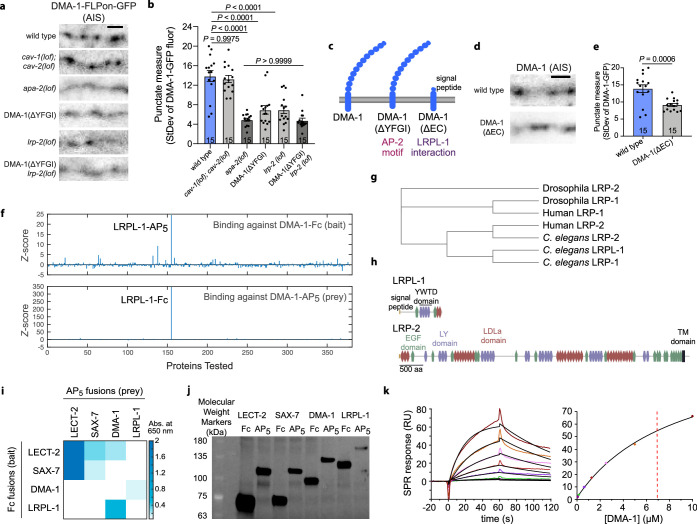
LRPL-1 interacts with the DMA-1 extracellular domain. (**a**) Cell-specific endogenous DMA-1-FLPon-GFP localization in the AIS of the indicated animals. DMA-1(ΔYFGI) is a genomic deletion of the putative AP-2-binding motif YXXΦ. (**b**) DMA-1 punctate measure of animals described in **a**. (**c**) Schematic of DMA-1 endocytic motifs. (**d**) Localization of wild type and extracellular domain deletion (ΔEC) versions of DMA-1-GFP. (**e**) Punctate measure of animals described in **d**. (**f**) Z-scores (signal defined as standard deviations over mean value of measurements) in the extracellular interactome assay (ECIA) for 380 *C. elegans* ectodomains as prey against Fc-tagged DMA-1 as bait (top), and Z-scores DMA-1-AP_5_ as prey (bottom). See Supplementary Table 1 for raw data. (**g**) Phylogenetic tree analysis created using Clustal Omega multiple sequence alignments. (**h**) Protein domain structures: LY repeats are indicative of LRP-type beta-propeller domains, known as YWTD domains. (**i**) Protein binding between four ectodomains using ECIA. Weaker DMA-1-Fc binding to LRPL-1-AP5 is due to LRPL-1-AP_5_ lower expression (see Extended Data Fig. 5j). (**j**) Western blot analysis of protein purification for ECIA experiments in Extended Data Fig. 5i. Western blot was to confirm protein expression and thus not repeated. Gel source data in Supplementary Fig. 1. (**k**) Surface plasmon resonance experiment for an LRPL-1-immobilized Biacore Streptavidin chip against DMA-1 ectodomain as analyte. Left: Blank-subtracted SPR sensorgrams for DMA-1 at 78 nM to 10 µM with kinetic model fits shown in black. Right: Binding isotherm for the SPR data fit to the Langmuir 1:1 binding model. The estimated *K*_D_, 6.9 ± 0.7 µM (±: standard error of the fit), is depicted with a red dashed line. All data are shown as mean ± s.e.m. *N*-values are indicated on bar graphs and represent the number of animals. *P* values in **b** were calculated using a two-way ANOVA with Šidák multiple comparison test. *P* values in **e** were calculated using a two-tailed unpaired *t* test. Scale bars, 2 µm (**a**, **d**). Source data

## Data Availability

All reagents and raw data are available from the corresponding author upon reasonable request. Source data are provided with this paper.

## Code availability

The MATLAB programs that were used to analyse the pulsed-pH data have been previously described^40,42^ and were written for MATLAB2018a with the following MATLAB toolboxes: Image Processing, Wavelet, Statistics and Machine Learning; the programs are formatted as a toolbox, scission_analysis, available at MATLAB Central File Exchange as 72744-scission_analysis (https://fr.mathworks.com/matlabcentral/fileexchange/72744-scission_analysis).
