# Author Correction: Endocytosis in the axon initial segment maintains neuronal polarity

**DOI:** 10.1038/s41586-025-08837-y

**Published:** 2025-03-18

**Authors:** Kelsie Eichel, Takeshi Uenaka, Vivek Belapurkar, Rui Lu, Shouqiang Cheng, Joseph S. Pak, Caitlin A. Taylor, Thomas C. Südhof, Robert Malenka, Marius Wernig, Engin Özkan, David Perrais, Kang Shen

**Affiliations:** 1 Howard Hughes Medical Institute, Department of Biology, Stanford University, Stanford, CA USA; 2 Department of Pathology, Stanford University School of Medicine, Stanford, CA USA; 3 Institute for Stem Cell Biology and Regenerative Medicine, Stanford University School of Medicine, Stanford, CA USA; 4 University of Bordeaux, CNRS, Interdisciplinary Institute for Neuroscience, Bordeaux, France; 5 Department of Molecular and Cellular Physiology, Stanford University, Stanford, CA USA; 6 Howard Hughes Medical Institute, Stanford University School of Medicine, Stanford, CA USA; 7 Nancy Pritzker Laboratory, Department of Psychiatry and Behavioral Sciences, Stanford University, Stanford, CA USA; 8 Department of Biochemistry and Molecular Biology, University of Chicago, Chicago, IL USA; 9 Grossman Institute of Neuroscience, Quantitative Biology and Human Behavior, University of Chicago, Chicago, IL USA

**Keywords:** Cellular neuroscience, Cell polarity, Membrane trafficking, Molecular neuroscience

Correction to: *Nature* 10.1038/s41586-022-05074-5 Published online 17 August 2022

In the version of the article initially published, Fig. 3j and Extended Data Fig. 5a contained errors that were generated during figure assembly. In both cases, the initially published accompanying source data file contains the correct data. None of the paper’s interpretations are affected by this correction.

Figure 3j contained an error in the *lrpl-1(lof) lrp2(lof)* mutant data, which were mistakenly graphed and inadvertently duplicated from the overexpressed DMA-1 condition of Fig. 1p. This error only pertains to the *lrpl-1(lof) lrp2(lof)* mutant data. Data for wild type and *degt-1(lof)* were intentionally re-graphed between Fig. 1p and Fig. 3j as specified in the published Fig. 3 legend. The corrected graph is shown below in Fig. 1, together with the incorrect published graph, for transparency to the readers. This error did not affect any conclusions because the source data were correct, and the mistaken bar graph in question was from a mutant phenotype with a very similar defect (76.67% vs 78.33% of animals responding).

**Fig. 1:**
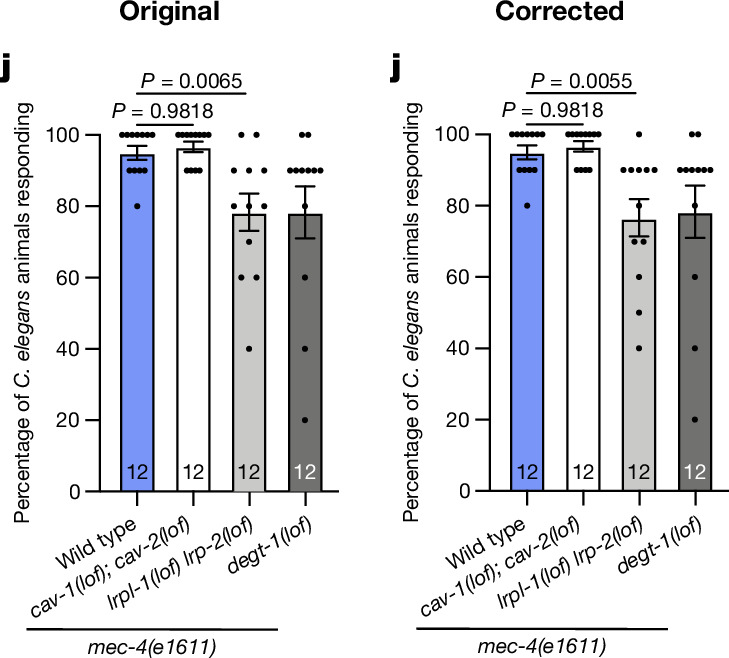
Original and corrected Fig. 3j .

Extended Data Fig. 5a contained an incorrect display micrograph for the DMA-1(ΔYFGI) mutant. This error occurred during figure assembly, and the corresponding quantification and raw data for this experiment, which are graphed in Extended Data Fig. 5b, are correct. The corrected figure panel is shown below in Fig. 2, together with the incorrect published display panel, for transparency to the readers. This error did not affect any conclusions because the source data were correct, and the mistaken display panel was from a statistically indistinguishable genotype.

**Fig. 2:**
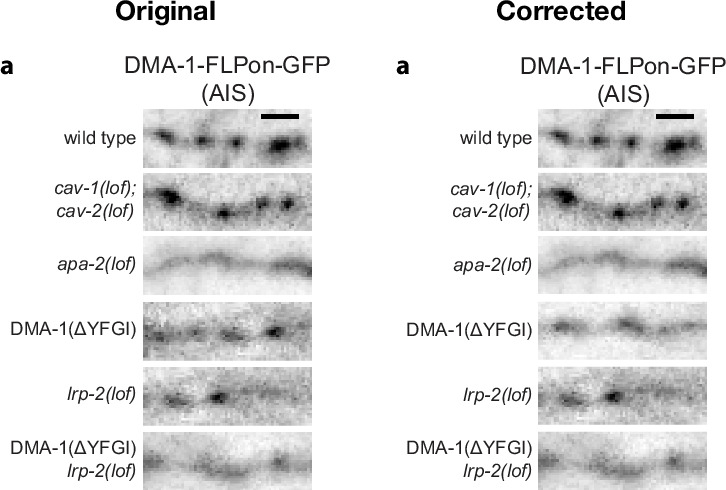
Original and corrected Extended Data Fig. 5a .

