# Supplementary material for: Endocytosis in the axon initial segment maintains neuronal polarity
Source: Nature. 2022 Aug 17;609(7925):128–35. doi: 10.1038/s41586-022-05074-5 (PMC9433327; doi:10.1038/s41586-022-05074-5)
Supplement: Supplementary file 1 — This file contains a Supplementary Note, Supplementary Fig. 1, legends for Supplementary Tables 1–4 and Supplementary Videos 1–6, and Supplementary References [file 41586_2022_5074_MOESM1_ESM.pdf]

nature portfolio

https://doi.org/10.1038/s41586-022-05074-5

Supplementary information

Endocytosis in the axon initial segment maintains neuronal polarity

In the format provided by the authors and unedited

Table of Contents

3 **Supplementary Note**

7 **Supplementary References**

8 **Supplementary Figure 1**

9 **Supplementary Table 1-4** (captions included, tables in Excel files)

10 **Supplementary Videos 1-6** (captions included, videos in .mov files online)

Supplementary Note 1

Molecular mechanism of DMA-1 endocytosis in the AIS

We hypothesized that elucidating DMA-1 endocytic mechanisms in the AIS would provide molecular access to preferentially perturbing endocytosis at the AIS. We first investigated the molecular mechanism of DMA-1 endocytosis through mutational analysis. Loss of function mutation in the clathrin-mediated endocytic adapter protein *apa-2* resulted in a redistribution of DMA-1 from punctate structures to a diffuse localization pattern in the AIS (Extended Data Fig. 5a-c). This is consistent with inhibited DMA-1 endocytosis and the loss of punctate structures that we observed with a *dynamin-1* temperature sensitive mutation (Fig. 2g, h). We then used a structure-function approach to identify two interaction motifs within DMA-1 that enable its efficient clathrin-mediated endocytosis in the AIS. We started with deletion of DMA-1's putative AP-2 binding motif in its cytoplasmic tail (residues YFGI)[33]. When this motif was endogenously deleted, DMA-1's punctate localization in the AIS was reduced (Extended Data Fig. 5a-c), thus supporting an intracellular motif that promotes DMA-1 endocytosis through a canonical AP-2 binding pathway. We then investigated if DMA-1's extracellular domain contributes to its endocytosis. Indeed, deletion of DMA-1's extracellular domain also caused a partial reduction in its punctate distribution in the AIS (Extended Data Fig. 5c-e), which suggests an extracellular interaction with DMA-1 that also facilitates its endocytosis.

To identify proteins that promote DMA-1 endocytosis through its extracellular domain, we performed an extracellular interactome assay, a high-throughput pairwise ectodomain interaction screen[65]. This screen of 380 *C. elegans* proteins from common neuronal cell surface receptor families identified an uncharacterized secreted low-density lipoprotein (LDL) receptor-like protein as the strongest DMA-1 binding partner (Extended Data Fig. 5f, Supplementary Table 1). We named this protein LRPL-1 (LRP-1 like; Extended Data Fig. 5g-k) based on its homology to LDL receptor-related protein 1 (LRP-1). The LRP family is a conserved family of proteins that function as endocytic co-receptors through extracellular domain interactions[67]. The *C. elegans* genome contains two transmembrane LRPs, LRP-1 and LRP-2, in addition to LRPL-1. We focused on LRPL-1 and LRP-2, which are close homologs of *C. elegans* LRP-1 and human LRP-2, respectively (Extended Data Fig. 5g).

Based on canonical LRP protein function as endocytic co-receptors, we tested if LRP proteins have a role in DMA-1 endocytosis. We examined both endogenous DMA-1 localization and GFP fluorescence in the DMA-1 cell surface reporter assay, which enables identification of puncta containing endocytosed DMA-1. Indeed, an *lrp-2* loss of function mutation changed the localization pattern of endogenous DMA-1 in the AIS and caused a decrease in DMA-1 puncta there (Extended Data Fig. 5a, b). This data suggests a role for LRP-2 in promoting DMA-1 endocytosis in the AIS. We further tested this idea using the DMA-1 cell surface reporter. This assay revealed a decrease in the GFP signal in the AIS, but not dendrite, in *lrp-2* and *lrpl-1* loss of function mutant animals (Extended Data Fig. 6a, b). Double mutation of *lrp-2* and *lrpl-1* did not enhance the defect, indicating that they function together in the same pathway (Extended Data Fig. 6a, b). Together, these results suggest that LRPL-1 and LRP-2 function to promote DMA-1 endocytosis preferentially in the AIS.

To further investigate LRP protein function, we next determined their localization. Because LRPL-1 is a secreted protein, based on domain predictions and confirmed by its successful secretion in our *in vitro* purification, we used an endogenous SL2 transcriptional reporter that identified the PVD neuron as a site of its transcription (Extended Data Fig. 6c). We then examined LRPL-1-GFP protein localization and found that LRPL-1-GFP localized to punctate structures in the AIS that colocalized with DMA-1 puncta (Extended Data Fig. 6d-f). Because DMA-1 puncta rarely enter the AIS (Extended Data Fig. 1f, g) and predominantly contain DMA-1 receptors that have been exposed to the cell surface (Fig. 2c, d), these puncta likely represent endocytic structures and not biosynthetic vesicles. Transmembrane LRP-2 was enriched in the AIS compared to the axon and dendrite (Extended Data Fig. 6g, h). LRP-2 also formed discrete puncta that were dependent upon the function of endocytic clathrin-coated pit associated proteins AP-2 and DAB-1 (the *C. elegans* homolog of DAB-2, LRP-2's known endocytic adapter protein[68,69]; Extended Data Fig. 6i-l). These results indicate that LRP-2 is concentrated at endocytic structures, which is consistent with its canonical function as an endocytic co-receptor.

We then performed experiments to determine how LRPL-1 and LRP-2 are functioning in DMA-1 endocytosis. LRP proteins promote the endocytosis of other transmembrane receptors when linked through binding a secreted protein[70]. Therefore, we considered the possibility of an endocytic complex of DMA-1, LRPL-1, and LRP-2 (Extended Data Fig. 6i) that functions in the AIS based on the following pieces of data: 1) DMA-1 interacts with secreted LRPL-1 through its extracellular domain (Extended Data Fig. 5f-k), 2) transmembrane LRP-2 and secreted LRPL-1 function in the same pathway to promote DMA-1 endocytosis in the AIS (Extended Data Fig. 6a, b), 3) both LRPL-1 and LRP-2 form puncta in the AIS (Extended Data Fig. 6d, e, g, h), and 4) LRPL-1 and DMA-1 colocalize in the AIS (Extended Data Fig. 6f). A prediction of this model is that LRPL-1's punctate localization to the AIS will require both AP-2 to be concentrated into endocytic clathrin-coated pits and internalized into the PVD neuron as well as its binding partner DMA-1 and LRP-2. Indeed, LRPL-1's localization to the AIS is dependent upon AP-2, DMA-1, and LRP-2 (Extended Data Fig. 6m-o). The requirement of AP-2 for LRPL-1 localization to the AIS supports the idea that these LRPL-1 puncta represent endolysosomal structures. These results are consistent with secreted LRPL-1 being internalized via clathrin-mediated endocytosis through interaction with DMA-1 and LRP-2.

Together, these findings support a working model in which DMA-1 is efficiently endocytosed in the AIS by engaging two pathways: 1) an intracellular interaction between DMA-1 and AP-2, and 2) an extracellular interaction between DMA-1 and LRP endocytic co-receptors (Extended Data Fig. 6i). Indeed, inhibition of both endocytic pathways through deletion of DMA-1's putative AP-2 motif (ΔYFGI) in combination with the loss of *lrp-2* phenocopied *apa-2* loss of function (Extended Data Fig. 5a, b). Therefore, we propose that DMA-1 has an additional endocytic module at the AIS through forming an endocytic co-receptor complex with LRPL-1 and LRP-2. The preferential function of this co-receptor complex at the AIS is supported by finding a specific effect of the LRP proteins at the AIS using the DMA-1 cell surface reporter assay and the subcellular enrichment of LRPL-1 and LRP-2 at the AIS. Together, these results suggest that LRP proteins preferentially promote DMA-1 endocytosis in the AIS, and thus provide molecular access to perturbing DMA-1 endocytosis at the AIS.

Preferential inhibition of DMA-1 endocytosis in the AIS through impairment of LRP proteins

The analysis of DMA-1 endocytic mechanisms provided molecular access to perturb DMA-1 endocytosis in the AIS through manipulation of the DMA-1 endocytic module that is preferential to the AIS. Indeed, disruption of this complex through deletion of DMA-1's extracellular domain or introduction of an *lrp-2* loss of function mutation resulted in DMA-1 mislocalization to the axon and a partial loss of DMA-1 dendritic polarity (Fig. 3f, g and Extended Data Fig. 6p, q). Combining these loss of function perturbations did not exacerbate the DMA-1 loss of polarity, suggesting that *lrp-2* functions through DMA-1's extracellular domain (Extended Data Fig. 6p, q). A loss of function mutation in *dab-1*, LRP-2's endocytic adapter protein, also resulted in DMA-1 mislocalization to the axon and a partial loss of DMA-1 dendritic polarity (Fig. 3f, g and Extended Data Fig. 6p-s). However, loss of function mutation in *lrp-2* did not affect the dendritic polarity of HPO-30, another dendritically polarized receptor, thus suggesting some specificity of LRP-2 function with DMA-1 (Extended Data Fig. 6t, u). These results suggest that DMA-1 endocytosis at the AIS through LRP-2 function is important for maintaining its polarity. Consistent with this, both *lrp-2* and *lrpl-1* mutation caused the axon to gain aberrant axonal branches similar to endocytic inhibition through *apa-2* mutation (Extended Data Fig. 6v, w & Fig. 3h, i), demonstrating a loss of morphological polarity. Additionally, mutation of *lrp-2* and *lrpl-1* caused behavioral deficits in a harsh touch escape behavioral assay, indicative of decreased neuronal function (Fig. 3j). Altogether, these results support the idea that endocytosis in the AIS contributes to DMA-1's dendritic polarity.

Supplementary References

33. Traub, L. M. Sorting it out: AP-2 and alternate clathrin adaptors in endocytic cargo selection. *Journal of Cell Biology* **163**, 203–208 (2003).
64. Cheng, S., Ashley, J., Kurleto, J. D., Lobb-Rabe, M., Park, Y. J., Carrillo, R. A. & Özkan, E. Molecular basis of synaptic specificity by immunoglobulin superfamily receptors in Drosophila. *Elife* **8**, (2019).
65. Özkan, E., Carrillo, R. A., Eastman, C. L., Weiszmann, R., Waghray, D., Johnson, K. G., Zinn, K., Celniker, S. E. & Garcia, K. C. An extracellular interactome of immunoglobulin and LRR proteins reveals receptor-ligand networks. *Cell* **154**, 228 (2013).
66. Chen, C. C. H., Schweinsberg, P. J., Vashist, S., Mareiniss, D. P., Lambie, E. J. & Grant, B. D. RAB-10 is required for endocytic recycling in the Caenorhabditis elegans intestine. *Mol. Biol. Cell* **17**, 1286–1297 (2006).
67. Spuch, C., Ortolano, S. & Navarro, C. LRP-1 and LRP-2 receptors function in the membrane neuron. Trafficking mechanisms and proteolytic processing in alzheimer's disease. *Frontiers in Physiology* (2012). doi:10.3389/fphys.2012.00269
68. Holmes, A., Fiett, A., Coudreuse, D., Korswagen, H. C. & Pettitt, J. C. elegans disabled is required for cell-type specific endocytosis and is essential in animals lacking the AP-3 adaptor complex. *J. Cell Sci.* **120**, 2741–2751 (2007).
69. Morris, S. M. & Cooper, J. A. Disabled-2 Colocalizes with the LDLR in Clathrin-Coated Pits and Interacts with AP-2. *Traffic* **2**, 111–123 (2001).
70. Jiang, Y., He, X. & Howe, P. H. Disabled-2 (Dab2) inhibits Wnt/β2-catenin signalling by binding LRP6 and promoting its internalization through clathrin. *EMBO J.* **31**, 2336–2349 (2012).

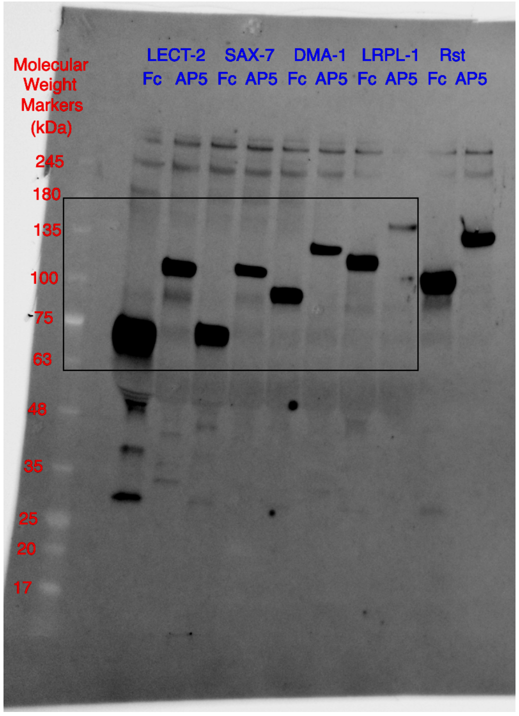

Supplementary Figure 1. Uncropped Western blots with molecular weight markers and indication of cropped region. Uncropped western blot blotted with an anti-His Tag antibody (THE™ His Tag Antibody [iFluor 488], mAb, Mouse, GenScript Cat# A01800) with molecular weight markers. Cropped region is indicated by the black box outline.

Supplementary Table Descriptions

Supplementary Table 1

Results from extracellular interactome assay presented in Extended Data Fig. 5f.

Supplementary Table 2

Description of *C. elegans* strains used in this study.

Supplementary Table 3

Description of constructs generated for this study.

Supplementary Table 4

Description of sgRNAs used to generate new alleles using CRISPR genome editing.

Supplementary Video Descriptions

Video S1. Dendritic protein vesicle dynamics in the dendrite.

Endogenous DMA-1-GFP vesicles are robustly trafficked in the dendrite. Scale bar, 2 μm.

Video S2. Dendritic protein vesicle dynamics in the AIS.

Endogenous DMA-1-GFP vesicles in the AIS are largely immobile (cell body/dendrite on left). Scale bar, 2 μm.

Video S3. Clathrin-labeled vesicle dynamics in the AIS.

Cell-specific endogenous GFP-FLPon-CLIC-1 puncta dynamics in the AIS showing stable endocytic structures as well as a mobile transport vesicle (dendrite on top). Scale bar, 1 μm.

Video S4. AP-2-labeled vesicle formation and disappearance in the AIS.

Cell-specific endogenous AP-2-FLPon-GFP puncta formation and disappearance in the AIS. Scale bar, 1 μm.

Video S5. Dendritic protein vesicle dynamics in the dendrite of *apa-2* mutant animals.

Cell-specific endogenous DMA-1-FLPon-GFP vesicles are robustly trafficked in the dendrite of *apa-2* mutants. Scale bar, 1 μm.

Video S6. Dendritic protein vesicle dynamics in the axon of *apa-2* mutant animals.

Cell-specific endogenous DMA-1-FLPon-GFP is diffuse and not punctate in the axon of *apa-2* mutants. Scale bar, 1 μm.
