# Supplementary material for: Endocytosis in the axon initial segment maintains neuronal polarity
Source: Nature. 2022 Aug 17;609(7925):128–35. doi: 10.1038/s41586-022-05074-5 (PMC9433327; doi:10.1038/s41586-022-05074-5)
Supplement: Supplementary file 2 — Reporting Summary [file 41586_2022_5074_MOESM2_ESM.pdf]

Corresponding author(s): Kang Shen

Last updated by author(s): May 30, 2022

Reporting Summary

Nature Research wishes to improve the reproducibility of the work that we publish. This form provides structure for consistency and transparency in reporting. For further information on Nature Research policies, see our Editorial Policies and the Editorial Policy Checklist.

Statistics

For all statistical analyses, confirm that the following items are present in the figure legend, table legend, main text, or Methods section.

n/a	Confirmed	
☐	☒	The exact sample size (*n*) for each experimental group/condition, given as a discrete number and unit of measurement
☐	☒	A statement on whether measurements were taken from distinct samples or whether the same sample was measured repeatedly
☐	☒	The statistical test(s) used AND whether they are one- or two-sided<br>*Only common tests should be described solely by name; describe more complex techniques in the Methods section.*
☒	☐	A description of all covariates tested
☐	☒	A description of any assumptions or corrections, such as tests of normality and adjustment for multiple comparisons
☐	☒	A full description of the statistical parameters including central tendency (e.g. means) or other basic estimates (e.g. regression coefficient) AND variation (e.g. standard deviation) or associated estimates of uncertainty (e.g. confidence intervals)
☐	☒	For null hypothesis testing, the test statistic (e.g. *F*, *t*, *r*) with confidence intervals, effect sizes, degrees of freedom and *P* value noted<br>*Give P values as exact values whenever suitable.*
☒	☐	For Bayesian analysis, information on the choice of priors and Markov chain Monte Carlo settings
☒	☐	For hierarchical and complex designs, identification of the appropriate level for tests and full reporting of outcomes
☒	☐	Estimates of effect sizes (e.g. Cohen's *d*, Pearson's *r*), indicating how they were calculated

Our web collection on statistics for biologists contains articles on many of the points above.

Software and code

Policy information about availability of computer code

Data collection	3i Slidebook v6 and MetaMorph versions 7.8.4.0, 7.8.12.0 and 7.10.3.279 were used to collect microscopy images.
Data analysis	GraphPad Prism v8 was used for statistical analyses. Fiji (ImageJ) v.2.1.0 and MATLAB 2018a and R2018b were used for image analysis. Custom MATLAB code used for analysis was previously published and publicly available (https://www.mathworks.com/matlabcentral/fileexchange/72744-scission_analysis). Biacore X100 Evaluation software version 2.0.2 to analyze sensorgrams.

For manuscripts utilizing custom algorithms or software that are central to the research but not yet described in published literature, software must be made available to editors and reviewers. We strongly encourage code deposition in a community repository (e.g. GitHub). See the Nature Research guidelines for submitting code & software for further information.

Data

Policy information about availability of data

All manuscripts must include a data availability statement. This statement should provide the following information, where applicable:

- Accession codes, unique identifiers, or web links for publicly available datasets
- A list of figures that have associated raw data
- A description of any restrictions on data availability

All data generated or analyzed during this study are included in this published article (and its supplementary information files).

Field-specific reporting

Please select the one below that is the best fit for your research. If you are not sure, read the appropriate sections before making your selection.

☒ Life sciences ☐ Behavioural & social sciences ☐ Ecological, evolutionary & environmental sciences

For a reference copy of the document with all sections, see nature.com/documents/nr-reporting-summary-flat.pdf

Life sciences study design

All studies must disclose on these points even when the disclosure is negative.

Sample size	No power analyses were used to predetermine the number of animals. Suitable sample sizes were determined based on previous experience and based on published literature in the field. 9-30 animals or cells were used for each condition. All sample sizes are specified in figures and legends (individual dots represent independent biological replicates, figure legends and/or methods).
Data exclusions	No data were excluded
Replication	Biological replicates are defined as distinct animals for C. elegans experiments or distinct neurons for cell culture experiments. All C. elegans in vivo microscopy measurements were repeated in at least two independent imaging sessions. All vertebrate neuron experiments were from at least 3 independent neuronal cultures and at least three independent imaging experiment sessions. All attempts at replication were successful. Experiments with in vitro purified proteins were not repeated due to the scale of the experiments and resulting resource requirements and because in vitro results were independently verified with subsequent in vivo experiments.
Randomization	Randomization was not necessary for these experiments. Experiments were performed based on genotype/treatment, and animals/neurons were not further separated into experimental sub-groups requiring randomization.
Blinding	Investigators were blinded to the genotype of animals for all behavioral assays. Otherwise, experiments were not blinded because mutant/ treatment phenotypes were easily observable and did not effectively allow for this.

Reporting for specific materials, systems and methods

We require information from authors about some types of materials, experimental systems and methods used in many studies. Here, indicate whether each material, system or method listed is relevant to your study. If you are not sure if a list item applies to your research, read the appropriate section before selecting a response.

Materials & experimental systems

n/a	Involved in the study	
☐	☒	Antibodies
☐	☒	Eukaryotic cell lines
☒	☐	Palaeontology and archaeology
☐	☒	Animals and other organisms
☒	☐	Human research participants
☒	☐	Clinical data
☒	☐	Dual use research of concern

Methods

n/a	Involved in the study	
☒	☐	ChIP-seq
☒	☐	Flow cytometry
☒	☐	MRI-based neuroimaging

Antibodies

Antibodies used

chicken polyclonal anti-MAP2, Abcam, Cat# ab5392
rabbit polyclonal anti-MAP2 Abcam Cat# ab32454
goat polyclonal anti-MAP2A/B EnCor Biotech Cat # GPCA-MAP2A/B
mouse anti-ankyrinG (clone N106/36) Sigma Cat# MABN466
Ankyrin G Polyclonal rabbit purified antibody Synaptic Systems Cat# 386 003
Polyclonal Guinea pig purified antibody Ankyrin G Synaptic Systems Cat# 386 005
rabbit polyclonal anti-clathrin heavy chain Abcam Cat# ab21679
goat polyclonal anti-AP-2 complex subunit alpha-1 Abcam Cat# ab189995
mouse anti-alpha adaptin monoclonal antibody (clone AP6) Thermo Cat# MA1-064
recombinant anti-RAB-7 antibody [EPR7589] Abcam Cat# ab137029
IHC-plus™ Polyclonal Goat anti-Mouse RAB7A / RAB7 Antibody, LSBio Cat# LS-B13237-100
sheep polyclonal anti-TGN46 (human) BioRad Cat# AHP500G
chicken polyclonal anti-beta III tubulin Abcam Cat# ab41489
mouse anti-L1CAM (clone UJ127.11) Sigma Cat# L4543
Anti-TfR antibody (clone H68.4) Thermo Fisher Scientific Cat #13-6800, RRID AB_2533029
goat polyclonal anti-transferrin receptor Thermo Fisher Cat# A80-128A
rabbit anti-glutamate receptor 1 antibody, Sigma Cat# AB1504

	rabbit anti-FLAG antibody Millipore Sigma Cat# F7425<br>rabbit polyclonal anti-Goat IgG (H+L), Alexa Fluor 488 Thermo Fisher Cat# A27012<br>donkey polyclonal anti-mouse IgG (H+L) Alexa Fluor Plus 488 Thermo Fisher Cat# A32766<br>goat polyclonal anti-chicken IgY (H+L) Alexa Fluor 555 Thermo Fisher Cat# A21437<br>donkey polyclonal anti-goat IgG (H+L) Alexa Fluor Plus 555 Thermo Fisher Cat# A32816<br>THE™ His Tag Antibody [iFluor 488], mAb, Mouse, GenScript Cat# A01800<br>goat polyclonal anti-rabbit IgG (H+L) Alexa Fluor 555 Thermo Fisher Cat# A27039<br>donkey polyclonal anti-sheep IgG (H+L) Alexa Fluor 555 Thermo Fisher Cat# A21436<br>goat polyclonal anti-chicken IgY (H+L) Alexa Fluor 647 Thermo Fisher Cat# A21449<br>goat polyclonal anti-rabbit IgG (H+L) Alexa Fluor 647 Thermo Fisher Cat# A32733<br>DyLight™ 405 AffiniPure Donkey Anti-Chicken IgY (IgG) (H+L) Jackson Cat# 703-475-155<br>DyLight™ 405 AffiniPure Donkey Anti-Mouse IgG (H+L) Jackson Cat# 715-475-151<br>Alexa Fluor® 488 AffiniPure Donkey Anti-Rabbit IgG (H+L) Jackson Cat# 711-545-152<br>Alexa Fluor® 488 AffiniPure Donkey Anti-Goat IgG (H+L) Jackson Cat# 705-545-147<br>Alexa Fluor® 594 AffiniPure Donkey Anti-Rabbit IgG (H+L) Jackson Cat# 711-585-152<br>Alexa Fluor® 647 AffiniPure Donkey Anti-Mouse IgG (H+L) Jackson Cat# 715-605-151<br>Alexa Fluor® 647 AffiniPure Donkey Anti-Chicken IgY (IgG) (H+L) Jackson Cat# 703-605-155<br>Alexa Fluor® 647 AffiniPure Donkey Anti-Guinea Pig IgG (H+L) Jackson Cat# 706-605-148<br>Donkey anti-Goat IgG (H+L) Highly Cross-Adsorbed Secondary Antibody, Alexa Fluor Plus 647 Thermo Cat# A32849<br>Monoclonal mouse anti-Neurofascin antibody Antibodies Incorporated Cat # 75-172<br>chicken polyclonal anti-mScarlet antibody Synaptic Systems Cat #409006<br>anti-Mouse Alexa568 conjugate, Thermo Fisher Scientific, A11004, RRID AB_2534072<br>Alexa Fluor 568 Donkey Anti-mouse IgG (H+L) Thermo Fisher Scientific Cat# A10037<br>Alexa Fluor 594 conjugate anti-chicken antibody Thermo Fisher Scientific Cat# A11042<br>Alexa Fluor 647 conjugated anti-mouse antibody Thermo Fisher Scientific Cat# A21235
Validation	All antibodies used are standard commercial antibodies and validated by the respective manufacturer. No custom antibodies that required validation were generated.

Eukaryotic cell lines

Policy information about cell lines

Cell line source(s)	Male human embryonic stem cells (ESCs) line H1: WiCell Research Institute, Inc. (WA01)<br>Human embryonic kidney (HEK) 293T cells: ATCC (CRL-11268)<br>S2 cells: Drosophila Genome Resource Center, Bloomington, Indiana<br>High Five cells: Laboratory of K. Christopher Garcia, Stanford University
Authentication	ESCs line H1 were authenticated by GTW banding karyotype method, and only cells with normal 46, XY karyotype were used for experiments. HEK293T were authenticated by ATCC using STR. S2 and High Five cells were not authenticated as they were only used for purified protein production and secretion, which was confirmed successful.
Mycoplasma contamination	Mycoplasma contamination has been bested and confirmed negative for all cell lines used in this study.
Commonly misidentified lines<br>(See ICLAC register)	No commonly misidentified lines used.

Animals and other organisms

Policy information about studies involving animals; ARRIVE guidelines recommended for reporting animal research

Laboratory animals	N2 Bristol strain Caenorhabditis elegans were used. L4 hermaphrodites were used for imaging and behavioral analysis. Males and hermaphrodites were used to generate new strains via crossing.<br>Time pregnant Sprague-Dawley rats (Janvier Labs) were housed with ad libitum feeding at 18–22 °C ambient temperature with 50–70% humidity, and a 12 hour light-dark cycle. Every effort was made to minimize the number of animals used and their suffering. Embryonic day 18 rat embryos were used to prepare rat primary neuronal cultures.<br>Newborn (postnatal day 2) male and female wild type CD1 mice (Charles River, Cat#22) were used for induced human neuron experiments. Mice were housed at room temperature (68-72 degrees Fahrenheit) with 30-70% humidity, and a 12 hour light-dark cycle.<br>C57BL/6J mice were housed at room temperature and 40-60% humidity with a 12 hour light-dark cycle with free access to food and water ad libitum. Hippocampi were dissected from P0 neonatal male and female wild-type mice. All procedures complied with the animal care standards set forth by the National Institutes of Health (NIH) and were approved by the Stanford University Administrative Panel on Laboratory Animal Care.
Wild animals	No wild animals used.
Field-collected samples	No field-collected samples used.
Ethics oversight	All experiments requiring mice were approved by the administrative panel on laboratory animal care (APLAC, Stanford University). All procedures for experiments involving rats were in accordance with the European guide for the care and use of laboratory animals and approved by the ethics committee of Bordeaux University (CE50) and the French Ministry of Research.

Note that full information on the approval of the study protocol must also be provided in the manuscript.
